# A large language model for complex cardiology care

**DOI:** 10.1038/s41591-025-04190-9

**Published:** 2026-02-06

**Authors:** Jack W. O’Sullivan, Anil Palepu, Khaled Saab, Wei-Hung Weng, Daniel K. Amponsah, Evaline Cheng, Yong Cheng, Emily Chu, Yaanik Desai, Aly Elezaby, Muhammad Fazal, Tasmeen Hussain, Sneha S. Jain, Daniel Seung Kim, Roy Lan, Jiwen Li, Wilson Tang, Natalie Tapaskar, Victoria Parikh, Ryan Sandoval, Gabriella Spencer-Bonilla, Bryan Wu, Kavita Kulkarni, Philip Mansfield, Dale Webster, Juraj Gottweis, Joelle Barral, Mike Schaekermann, Ryutaro Tanno, S. Sara Mahdavi, Vivek Natarajan, Alan Karthikesalingam, Euan Ashley, Tao Tu

**Affiliations:** 1 Stanford University, Stanford, CA USA; 2 Google Research, Mountain View, CA USA; 3 Google DeepMind, Mountain View, CA USA

**Keywords:** Diagnosis, Medical research

## Abstract

The scarcity of subspecialist medical expertise poses a considerable challenge for healthcare delivery. This issue is particularly acute in cardiology, where timely, accurate management determines outcomes. We explored the potential of Articulate Medical Intelligence Explorer (AMIE), a large language model-based experimental medical artificial intelligence system, to augment clinical decision-making in this challenging context. We conducted a randomized controlled trial comparing large language model-assisted care with the usual care of complex patients suspected of having a genetic cardiomyopathy, and we curated a real-world dataset of complex cases from a subspecialist cardiology practice. Nine participating general cardiologists were provided with access to both clinical text reports and raw diagnostic data—including electrocardiograms, echocardiograms, cardiac magnetic resonance imaging scans and cardiopulmonary exercise testing—and were randomized to manage these cases, either with or without assistance from AMIE. We developed a ten-domain evaluation rubric used by three blinded subspecialists to evaluate the quality of triage, diagnosis and management. In our randomized controlled trial with retrospective patient data, subspecialists favored large language model-assisted responses overall, and for the management plan and diagnostic testing domains, with the remaining domains considered a tie. Overall, subspecialists preferred AMIE-assisted cardiology assessments 46.7% of the time, compared with 32.7% for cardiologists alone (*P* = 0.02), with 20.6% rated as a tie. Subspecialists also quantified errors, extra and missing content, reasoning and potential bias. Cardiologists alone had more clinically significant errors (24.3% versus 13.1%, *P* = 0.033) and more missing content (37.4% versus 17.8%, *P* = 0.0021) than cardiologists assisted by AMIE. Lastly, cardiologists who used AMIE reported that AMIE helped their assessment more than half the time (57.0%) and saved time in 50.5% of cases.

## Abstract (web summary)

In a randomized study involving 9 general cardiologists and 107 real-world patient cases, assistance from a specifically tailored large language model resulted in preferable responses on complex case management compared to physicians alone, as rated by specialist cardiologists using a multidimensional scoring rubric.

## Main

Globally, there is a substantial shortage of specialized medical expertise^1^. The World Health Organization predicts a deficit of 18 million providers by 2030, with shortages being most acute in resource-limited and rural areas^2^. This disparity is exacerbated for rarer and more complex conditions, particularly those for which timely treatment prevents morbidity and mortality. For instance, hypertrophic cardiomyopathy (HCM) is one of the leading causes of sudden cardiac death in young adults^3^, yet more than half of US states do not have an HCM subspecialist center^4^. This lack of subspecialist access has led to 60% of patients with HCM being undiagnosed in the USA^5^. Given that premature mortality is highly preventable with implanted cardiac defibrillators^3^, cardiac conditions such as HCM exemplify an urgent unmet need in healthcare delivery, namely timely and widely available access to subspecialist expertise^6^. While cardiac conditions serve as an indicative example, the consequences of delayed access to subspecialist care are profound across all specialties, often resulting in increased morbidity and mortality, as patients miss critical diagnostic and treatment windows. Navigating the cascade of referrals required to access subspecialist expertise creates undue stress and anxiety while presenting a time-consuming and resource-intensive process for both patients and healthcare providers.

Large language models (LLMs) have emerged as potential assistive tools for an array of healthcare issues^7,8^. LLMs can rapidly synthesize data from multiple sources and suggest differential diagnoses and management plans^9–11^. Some LLMs have already been integrated into electronic medical record software^12,13^ as assistive tools for summarization and communication. Despite the potential of LLMs to enhance medical expertise, rigorous assessment of their performance remains scarce in medical specialties, with few openly available datasets for model evaluation and almost no randomized controlled trials (RCTs) performed. It remains unclear whether LLMs possess the nuanced understanding and intricate knowledge base required to effectively replicate the decision-making process of experts in highly specialized medical fields^14^.

This study probes the potential of LLMs to democratize subspecialist-level expertise by focusing on an indicative example: the domain of genetic cardiomyopathies like HCM. We conducted an RCT in which cardiologists clinically assessed patients using comprehensive real-world clinical data with artificial intelligence (AI) assistance. This included both physician text reports and data for electrocardiograms (ECGs), transthoracic echocardiograms (TTEs), cardiac magnetic resonance imaging (CMR) scans and cardiopulmonary stress tests (CPX). Compared to earlier studies relying on simulated or text-only data, our design represents a substantial advancement toward real-world clinical applicability. We introduce an open-source dataset encompassing cardiac testing and genetic information from real-world patients at the Stanford Center for Inherited Cardiovascular Disease (SCICD), enabling further research in this specialized field. We use Articulate Medical Intelligence Explorer (AMIE)^9^, an LLM-based system built upon Gemini 2.0 Flash, to generate detailed assessments of patients with suspected complex cardiovascular disease. We propose detailed rubrics that subspecialists used to compare and evaluate the quality of diagnosis, triage and clinical management proposals for complex cardiology cases. Finally, we explore the potential of LLMs to upskill general cardiologists by evaluating whether interaction with AMIE improves their clinical decision-making. In our RCT, general cardiologists’ clinical assessments aided by AMIE were preferred overall, saved time in 50.5% of cases and had fewer clinically significant errors and fewer omissions of important content.

## Results

**Fig. 1:**
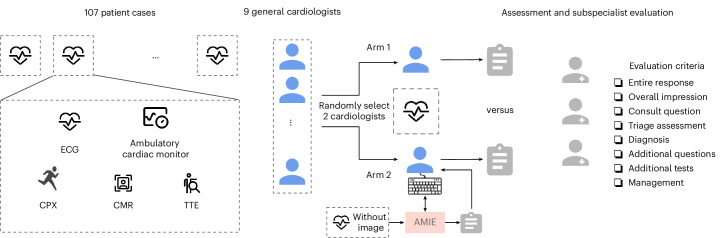
Study design. Text reports from the cardiac testing data of 107 patients with suspected genetic cardiovascular disease were provided to AMIE, and AMIE completed the assessment form listed in Extended Data Fig. 2. For the RCT, a pool of general cardiologists was randomized to either AI assistance or no assistance across the 107 cases, with each case being completed by two general cardiologists (one per arm). All general cardiologists had access to text reports from the cardiac testing data, as well as the raw multimodal artifacts. The general cardiologists in each arm completed the assessments and additional questions listed in Extended Data Fig. 2. In the arm with AI assistance, these cardiologists could view AMIE’s assessment and interact live with the system over a text-based chat interface (Extended Data Fig. 1). Blinded subspecialist cardiologists from SCICD provided individual ratings and direct preferences between the assessments produced from each arm using the forms listed in Extended Data Fig. 3. Subspecialists were blinded to the source of the ratings, and the assessments were presented in a randomized sequence.

**Extended Data Fig. 1:**
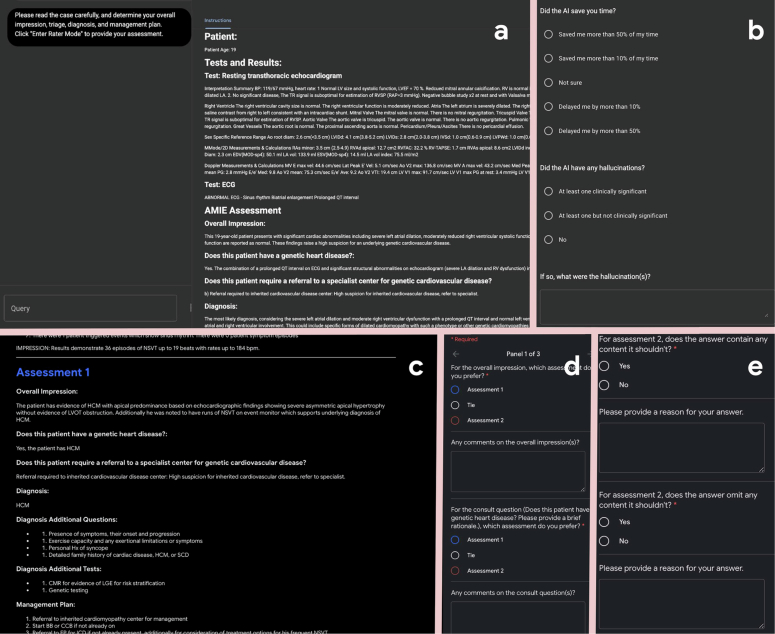
User interfaces for RCT. **a**) UI for AMIE assistance. This includes the case text, AMIE’s assessment, and a text-chat interface with AMIE. An ID is provided linking the case to the raw imaging from Stanford (AMIE/Google did not have access to this). **b**) Assessment form for general cardiologists. General cardiologists complete the assessment form listed in Extended Data Fig. 2. **c**) UI for subspecialist evaluators. This includes the case text and two assessments (one with AMIE-assistance, one without, in a randomized blinded sequence). **d**) Preference form for subspecialist evaluators. Subspecialist cardiologists rate their preference between the two assessments on the preference criteria in Extended Data Fig. 3. **e**) Individual assessment form for subspecialist evaluators. Subspecialist cardiologists rate each assessment on the set of individual criteria listed in Extended Data Fig. 3.

Figure 1 shows the study overview in which 107 consecutive patients were assessed by two general cardiologists, one with and one without AMIE assistance, using the interface shown in Extended Data Fig. 1. Details of the AMIE system are presented in Supplementary Section 1.

The median age of the patients was 59 years (range 18–96 years). The number and percentage of patients with available clinical text data for each test were as follows: CMR 64 (59.8%), CPX 65 (60.7%), resting TTE 90 (84.1%), exercise TTE 69 (64.5%), ECG 99 (92.5%), ambulatory Holter monitor 79 (73.8%) and genetic testing 77 (72.0%) (Table 1). Of the 107 patients, 39 (36.4%) had a variant adjudicated to be pathogenic or likely pathogenic as per the interpretation of variant criteria by the American College of Medical Genetics and Genomics^15^.

**Table 1:** Clinical text data availability across patients

	Number (%)
Median age	59.0 (range 18–96)
HCM	22 (20.6%)
Left ventricular noncompaction	21 (19.6%)
Dilated cardiomyopathy	8 (7.5%)
Arrhythmogenic cardiomyopathy	11 (10.3%)
Ischemic cardiomyopathy	11 (10.3%)
Other genetic	11 (10.3%)
Non-genetic/general	21 (19.6%)
CMR	64 (59.8%)
CPX	65 (60.7%)
Resting TTE	90 (84.1%)
Exercise TTE	69 (64.5%)
ECG	99 (92.5%)
Ambulatory Holter monitor	79 (73.8%)
Genetic testing (not used)	77 (72.0%)

The median age was 59 years (range 18–96 years) across the 107 test cases. Clinical text data span seven different modalities: CMR, CPX, resting TTE, exercise TTE, ECG, ambulatory Holter monitor and genetic testing. The genetic testing results were not provided to general cardiologists or AMIE during the RCT.

### General cardiologists’ perspectives on LLM use in clinical care

**Fig. 2:**
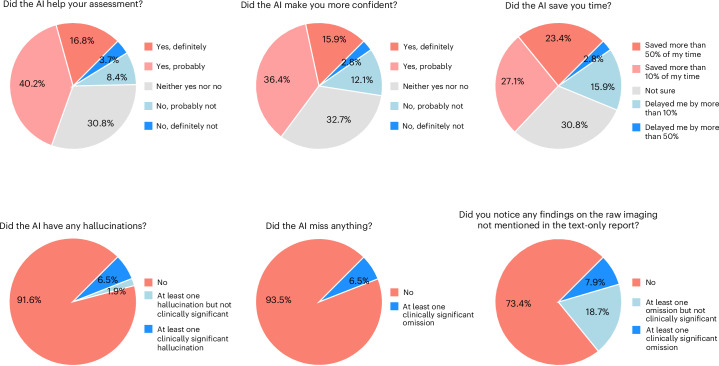
General cardiologist perspective on the use of AI. General cardiologists with access to AMIE answered additional questions after assessing the patients regarding their use of AI and the raw imaging data. These questions are listed in Extended Data Fig. 2. The pie charts display the proportions of chosen responses across the five questions related to AI use (*n* = 107) and the imaging question (*n* = 214).

**Extended Data Fig. 2:**
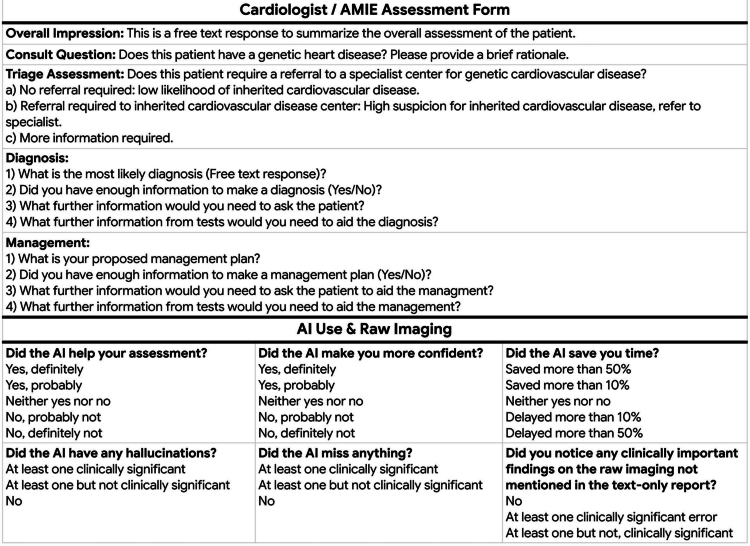
Assessment form for AMIE/cardiologist responses to cases. Prior to the randomized study, AMIE was provided with clinical text from various cardiac testings for each patient and asked to complete the top portion of the assessment form. During the RCT, general cardiologists had access to the clinical text from these cardiac testings as well as the raw imaging, and they completed the full assessment form. The ‘AI Use’ questions were only completed by the study arm that had access to AMIE.

As shown in Fig. 2, general cardiologists responding to the questions in Extended Data Fig. 2 expressed a favorable view of LLM integration into their clinical workflows. In a majority (57.0%) of cases, cardiologists reported that the AI improved their clinical assessments (16.8% “definitely” and 40.2% “probably”), while only 12.1% indicated it was unlikely to be helpful. Similarly, in 52.3% of cases, the general cardiologists stated that the AI increased their confidence in decision-making (15.9% “definitely” and 36.4% “probably”), while only 14.9% reported any decrease in confidence. With respect to efficiency, the general cardiologists indicated time savings by the AI in 50.5% of cases; notably, they reported saving more than 50% of their time in 23.4% of these cases. Only 18.7% of the cases noted any delays attributable to AI use, and the incidence of AI hallucinations was low. In 91.6% of cases, there were no reported hallucinations, while in 6.5% of cases, a likely clinically significant hallucination was observed. Similarly, in 93.5% of cases, the general cardiologists reported that the LLM did not miss anything (Fig. 2). Supplementary Section 2 presents the results for each cardiologist, and we further analyzed the free-text comments left by the general cardiologists in ‘Analysis of general cardiologist feedback’.

### Direct preference comparing general cardiologists with and without AMIE

**Fig. 3:**
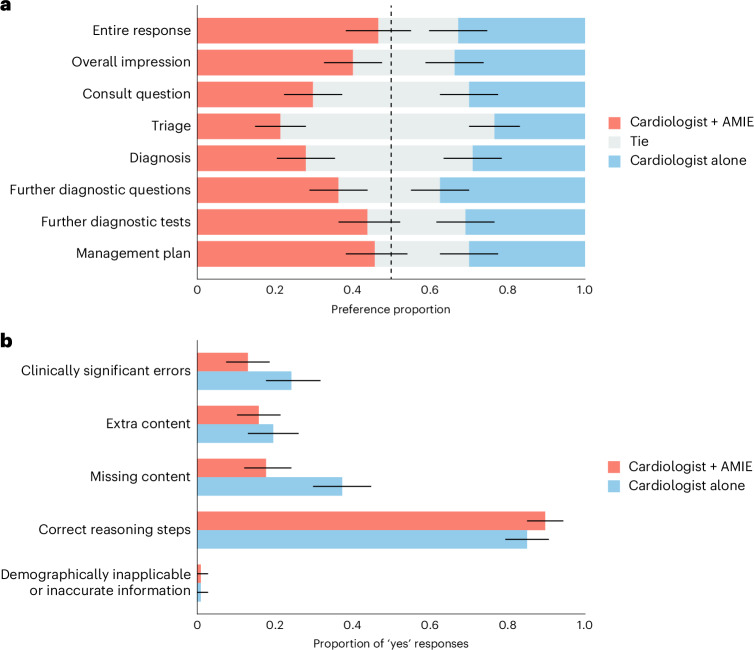
Subspecialist ratings. **a**, Preference between cardiologist + AMIE versus cardiologist alone. Subspecialists compared the two assessments per case and answered the preference questions shown in Extended Data Fig. 3 regarding their preference for each domain across the 107 paired assessments. They were blinded to the source of each assessment. Bars indicate, for each criterion, the proportion of subspecialist preference choices across the 107 scenarios, with error bars indicating 95% confidence intervals computed by bootstrapping the scenarios *n* = 10,000 times. The vertical dashed line represents the 50% threshold. **b**, Individual assessment of cardiologist + AMIE and cardiologist alone responses. For each of the 107 assessments from the two study arms, subspecialists answered the yes/no questions in Extended Data Fig. 3. Bars indicate, for each criterion, the proportion of ‘yes’ responses across the 107 scenarios, with error bars indicating 95% confidence intervals computed by bootstrapping the scenarios *n* = 10,000 times.

**Extended Data Fig. 3:**
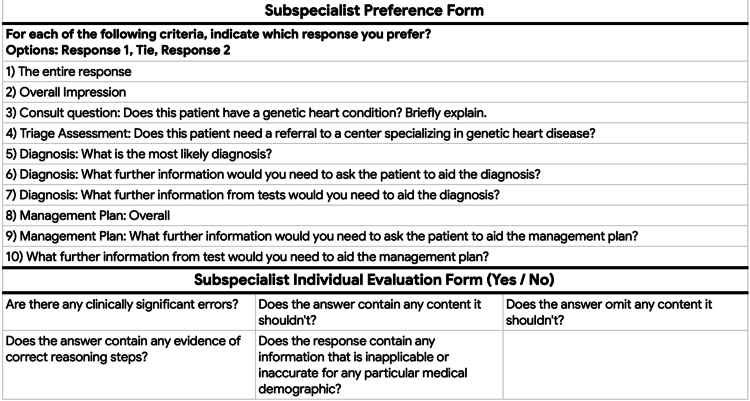
Subspecialist evaluation forms. First, subspecialists were provided with two different responses (blinded to which was produced by a cardiologist with AI assistance) and asked to supply their preference (Response 1, Tie, or Response 2) for 9 different aspects of the response as well as the entire response. Next, subspecialists independently answered each of these 5 questions (Yes/No) for each response. They were blinded to the source of each response when performing these evaluations.

A blinded evaluation by subspecialist cardiologists, responding to the questions in Extended Data Fig. 3, demonstrated a consistent pattern favoring LLM-assisted responses across several key clinical domains. For the entire clinical response, AMIE-assisted outputs were significantly preferred, with subspecialists preferring 46.7% of all cases compared to 32.7% for unassisted cardiologists (*P* = 0.02). For overall management, AMIE-assisted assessments were preferred for 45.8% of the cases compared to 29.9% for unassisted cardiologists (*P* = 0.008). Similarly, for the diagnosis domain related to further testing, AMIE-assisted responses were preferred for 43.9% of the cases compared with 30.8% for the unassisted (*P* = 0.03). General cardiologist responses alone were not preferred across any of the domains, although their responses were considered equivalent to the AMIE-assisted responses across the remaining domains, including consult question, triage, diagnosis and further diagnostic questions for the patient (Fig. 3).

### Individual assessment of general cardiologists’ responses with and without AMIE

The subspecialist cardiologists found that AMIE-assisted responses were either better or equivalent across clinically significant errors, extra content, missing content, clinical reasoning and bias.

The subspecialists also found that AMIE-assisted general cardiologist responses had significantly fewer errors: 13.1% of AMIE-assisted responses contained errors compared with 24.3% for unassisted responses (11.2% difference, *P* = 0.033). Similarly, there was significantly less missing content for AMIE-assisted responses: 17.8%, compared with 37.4% for unassisted responses (19.6% difference, *P* = 0.0021). There was no statistically significant difference in extra content between the responses, and both responses contained equivalent clinical reasoning steps and a lack of demographic bias (Fig. 3). The ‘Analysis of specialist feedback’ section shows an analysis of the free-text comments left by the subspecialists.

### Qualitative analysis

**Extended Data Fig. 4:**
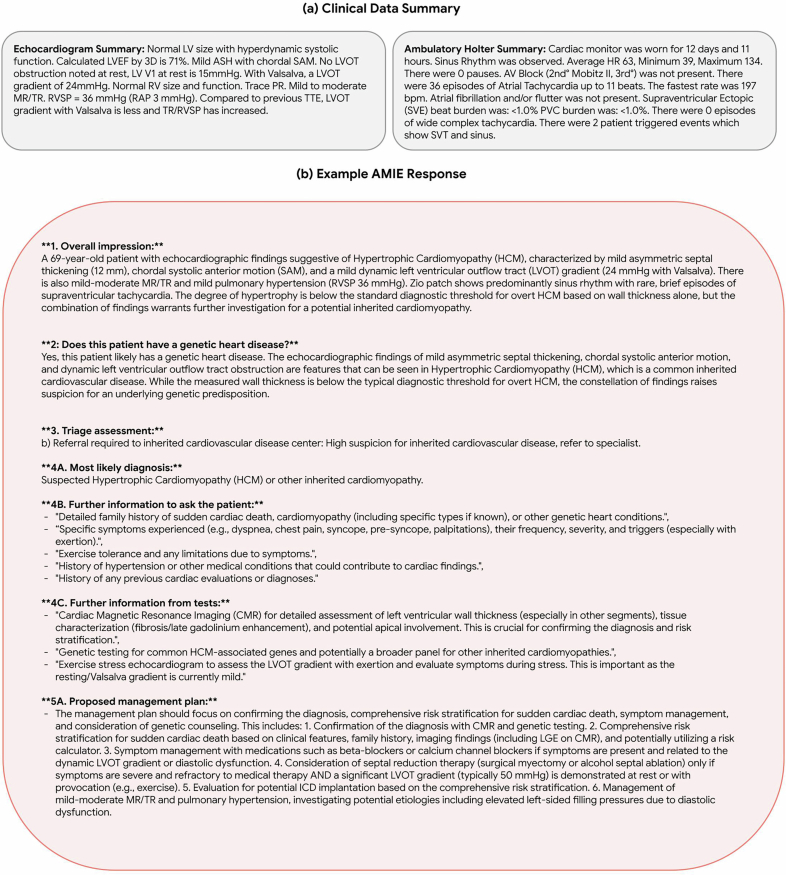
Example model response. **a**) Summaries of the clinical data provided to AMIE. **b**) The response provided by AMIE to the questions in Extended Data Fig. 2.

In Extended Data Fig. 4, we provide an example of AMIE’s standalone assessment. We also include several examples of how general cardiologists chose to interact with AMIE to aid in the production of their final assessments in Supplementary Section 3. These interactions with AMIE ranged from targeted querying for specific knowledge to more extensive engagement to work through disparate test results and build arguments for and against potential diagnoses and management plans.

During the study, general cardiologists were asked to provide comments on their use of AMIE, while subspecialists left comments on the quality of AI-assisted and unassisted cardiologist assessments. In addition to having a subspecialist cardiologist summarize this feedback, we used Gemini 2.5 Pro to analyze the feedback from the general cardiologists (summary in ‘Analysis of general cardiologist feedback’, automated report in Supplementary Section 4) and subspecialists (summary in ‘Analysis of specialist feedback’, automated report in Supplementary Section 5).

#### Analysis of general cardiologist feedback

The general cardiologists provided additional comments regarding their AI use for 41 out of the 107 cases. We used Gemini 2.5 Pro to categorize and summarize this feedback, with the prompt and full generated report shown in Supplementary Section 4. The described benefits include providing detailed information on rare conditions, provoking critical thinking and reconsideration of initial assessments, and saving time. Observed errors include the AI outputs being overly verbose, overconfident and/or dogmatic and occasionally lacking clinical nuance, and the inability to interpret temporal data properly.

The general cardiologists also documented instances of LLM hallucinations across eight cases, which fell into several categories: diagnostic assertions that contradicted the general cardiologist’s opinions, fabrication of imaging findings absent from source reports (for example, ‘left ventricular hypertrabeculation’), unwarranted assumptions regarding patient demographics, such as gender when such information was not provided, and misinterpretation of quantitative measurements, including confusion between exercise and resting aortic parameters. The identified errors ranged from subtle misinterpretations to more significant hallucinations, wherein AMIE generated medical information unsupported by the original text-imaging reports. Notably, these hallucinations were often amenable to correction when challenged by the cardiologists. For instance, when AMIE initially fabricated the presence of left ventricular hypertrabeculation, direct questioning by the general cardiologist prompted the system to self-correct and acknowledge that no abnormal trabeculation was present in the imaging report data.

The general cardiologists provided qualitative feedback on seven examples where the LLM missed clinical information (‘omissions’). These comments highlighted AMIE’s tendency to sometimes overlook or inadequately process existing diagnostic information, including failing to recognize that CMR and stress echocardiography had already been completed, providing insufficient detail about important findings such as regional wall motion abnormalities and trabeculations on echo, and missing critical historical information such as prior myocardial infarction mentioned in stress tests. Certain omissions resulted from AMIE’s lack of access to imaging test dates, leading to erroneous clinical interpretations. For instance, AMIE incorrectly concluded that discrepancies existed between left ventricular ejection fraction measurements obtained via CMR and TTE, asserting that one dataset contained errors. However, chart review by the general cardiologists revealed that both left ventricular ejection fraction measurements were accurate, with the apparent discrepancy attributable to a therapeutic intervention that occurred between the two studies—information that was not accessible to AMIE. Lastly, AMIE made redundant recommendations for tests already completed (such as ambulatory event monitoring). Overall, omissions were considered mild and minimal, in line with systematic quantitative feedback that showed AMIE did not miss anything in 93.4% of cases.

#### Analysis of specialist feedback

The subspecialist cardiologists provided 138 comments regarding their preference ratings between the assisted and unassisted responses, spanning 43 of the 107 cases. They also left specific comments on 55 of the 107 assessments where cardiologists had AMIE assistance and 69 of the 107 assessments without assistance, giving a total of 184 informative comments across the individual assessments. We used Gemini 2.5 Pro to categorize and summarize this feedback, with the prompt and full generated report shown in Supplementary Section 5.

In summary, the AI-assisted cardiologists were preferred more often, seemingly driven by their more comprehensive responses, fewer omissions and incorporation of modern diagnostics and advanced treatments. However, some responses were also flagged due to excessive detail, diagnostic overreach and occasional leaps in logic. The unassisted cardiologists were often praised for clear and concise reasoning but tended to have clinically significant omissions and errors related to key management decisions. Many of the cited types of errors, such as missing and/or incorrect diagnoses or screening and/or testing errors, were seen with similar frequencies in both study arms, suggesting that AI assistance did not have a consistent impact in those areas.

## Discussion

In this study, we probe the ability of LLMs to provide additive support to generalists in the assessment of rare, life-threatening cardiac diseases that typically require subspecialty cardiac care. Further, we address the unmet need for the randomized evaluation of LLMs for challenging medical applications. To this end, we curate an open-source, de-identified, real-world clinical dataset for patients suspected to have inherited cardiomyopathies and propose an evaluation rubric for the quality of diagnosis, triage and clinical management of such patients. Blinded subspecialists employed this evaluation rubric to assess clinical assessments performed by general cardiologists, both with and without LLM assistance. The blinded evaluation by the subspecialist cardiologists demonstrated an overall preference for LLM-assisted clinical assessments. Specifically, the subspecialty cardiologists found that AMIE-assisted clinical assessments demonstrated fewer clinically significant errors (11.2% reduction) and missed less important content (19.6% reduction) while maintaining equivalent clinical reasoning quality and not introducing erroneous extraneous information. Furthermore, general cardiologists who utilized AMIE reported that the system helped their assessments in more than half of cases (57.0%), did not miss clinically significant findings in 93.5% of cases and reduced assessment time in over half of cases (50.5%).

Our results demonstrate the feasibility of using LLMs to assess patients with rare and life-threatening cardiac conditions. Adapting AMIE to this subspecialist and rarified domain was highly data-efficient, leveraging iterative feedback from subspecialist experts to enhance the quality of AMIE’s responses using just nine cases. This iterative process, combined with a self-critique and the incorporation of search functionality, enabled AMIE to assist general cardiologists in upskilling their clinical assessments to a preferred level. This contrasts with earlier studies using generic, nonspecialized LLMs, which did not achieve comparable clinical performance^14^.

Our RCT results indicate that LLMs can assist general cardiologists in diagnosing and managing complex cardiac patients. Our evidence suggests that LLMs could help bridge unmet needs in genetic cardiovascular disease and possibly in cardiac care more broadly. While further research could extrapolate our approach to a broader group of specialties, cardiology is a useful indicative example because it features (a) highly preventable morbidity and mortality, (b) a reliance on an array of clinical investigations (for example, TTEs, ECGs, ambulatory Holter monitors, CMRs, CPXs and, when appropriate, genetic testing) and (c) a substantial deficit in the cardiology workforce. Our findings are particularly noteworthy because access to subspecialist care is a global challenge. The American College of Cardiology has identified a "cardiology workforce crisis," with the lack of access to subspecialty cardiologists an acute concern^6^. In the USA, despite five HCM centers of excellence in both California and New York, there are none across 27 states^4^. This has led to more than 60% of patients with HCM in the USA being undiagnosed, with estimates higher globally^5^. The propensity of inherited cardiomyopathy to cause sudden cardiac death (the leading cause of sudden cardiac death in young adults^3^), exacerbates the problem. Lack of access to appropriate care and long wait times can lead to preventable, premature mortality. LLMs may help identify undiagnosed cases, assist with the triage and prioritization of urgent cases, and streamline management. In this way, LLMs could improve access to specific care by assisting generalists.

For researchers, our results have a number of implications. First, we have made our data openly available, facilitating the rigorous evaluation of our results and providing data for other models to use in their tests. We have also created and validated a 10-domain evaluation rubric that may be used for future studies. More broadly, this study demonstrates the feasibility of conducting RCTs to evaluate LLMs, establishing a gold-standard evidence framework that should guide future research in this domain. Currently, LLMs are being used in many US health systems via their implementation in electronic medical records software^13^. This implementation has occurred without a similar scale of scientific evaluation; the benefits and the possible harms are only partially known^16^. These results represent a major step toward demonstrating the real-world utility of LLMs in subspecialty care.

The implications for clinicians are twofold. First, our results show a clear, albeit modest, improvement in overall clinical assessment quality. The significantly fewer errors and extra content, as well as the significant improvement in management plan quality, gives insights into how LLMs can assist clinically. The general cardiologists demonstrated high precision in diagnostic accuracy and triage decisions; however, the nuanced clinical management of complex patients was associated with increased omission errors compared to the AMIE-assisted assessments. These clinical improvements were accompanied by enhanced efficiency and increased clinician confidence. As such, we present RCT-level evidence for LLMs improving clinical care overall, specifically driven by improvements in management and a reduction in clinical errors and erroneous extra content, with simultaneous improvements in the time and confidence of providers.

It seems premature to deploy LLMs autonomously, and our RCT did not address this design directly, as general cardiologists reported that 6.5% of AMIE’s responses contained clinically significant hallucinations. Reassuringly, we showed that when LLMs are deployed with cardiologist oversight, hallucinations are most often identified, and this combination results in overall fewer errors and more preferred assessments. We qualitatively explored the nature of these hallucinations and found that the general cardiologists often described them as ‘mild’, ranging from assuming the patient’s sex to hallucinating the presence of a CMR feature (for example, “LLM stated left ventricular hypertrabeculation on CMR, but this is not explicitly stated”). Notably, the general cardiologists found that when asked about the hallucination, AMIE would correct itself.

Our study addresses a meaningful wider gap in earlier literature. Prior research has evaluated LLMs in a number of different settings in medicine, from assessing quality in question-answering (spanning medical license examinations as well as open-ended medical questions) to clinical image and complex diagnostic challenges^11,17–24^. There is a paucity of prior RCTs in medicine and cardiology. Despite more than 500 observational LLM papers published in 2024, systematic reviews of LLMs in medicine have consistently shown a lack of RCTs^25–27^. In fact, a 2025 systematic review found no RCTs assessing LLMs in cardiology^26^, while another concluded that “randomized trials or prospective real-world evaluations are needed to establish the clinical utility and safety of model use” and that “real-world trials addressing this possibility remain sparse”^28^. Beyond cardiology, there are some RCTs exploring LLMs. A 2024 JAMA Network Open study^29^ randomized physicians to use GPT-4 versus conventional resources alone for diagnostic reasoning on just six non-real-world clinical vignettes and found no significant improvement in diagnostic performance (76% versus 74%, *P* = 0.60), and no significant difference in time spent per case (519 versus 565 seconds, *P* = 0.20), but that GPT-4 alone outperformed both physician groups by 16 percentage points (*P* = 0.03). Similarly, a 2025 Nature Medicine RCT^30^ randomized physicians to use GPT-4 versus conventional resources for management reasoning tasks on five expert-developed clinical vignettes and found significant improvements in overall performance (6.5% improvement, *P* < 0.001), with management decisions improving by 6.1% and diagnostic decisions by 12.1%, demonstrating that LLMs may be more effective for treatment planning than initial diagnostic reasoning, in line with our results.

Observational studies have investigated the utility and potential of LLMs in real-world clinical tasks such as clinical letter generation^31^, medical information communication^32^, medical summarization^8,33^ and triaging mammograms and chest X-rays for tuberculosis^34^. Existing research on the performance of LLMs in medical subspecialties, such as cardiology^35^, ophthalmology^36^, gastroenterology^37^, neurology^35^ and surgery^38^, are also mostly limited to medical question-answering or examination benchmarking. A recent study^39^ evaluated ChatGPT in providing accurate cancer treatment recommendations concordant with authoritative guidelines with fixed question prompts. Another study investigated the diagnostic and triage accuracy of the GPT-3 relative to physicians and laypeople using synthetic case vignettes of both common and severe conditions^40^. A 2024 study^41^ compared GPT-4 performance with human experts in answering cardiology-specific questions from general users’ web queries. Our study is not only one of the first RCTs of LLMs in subspecialty domains^42^, it is also, to our knowledge, one of the first to use real-world data and to make this data for LLM evaluation available open-source.

The existing literature shows mixed results for LLMs in clinical cases^8,14^. A recent study showed the potential and safety concerns of using LLMs to provide an on-demand consultation service that assists clinicians’ bedside decision-making based on patient electronic health record data^43^. A 2024 study assessed the ability of LLMs to diagnose abdominal pathologies and showed that LLMs were inferior to clinicians^14^, though that study was not an RCT, and the authors noted that their results may be improved with fine-tuned LLMs. Although we did not fine-tune for this particular downstream task, our approach, which included using a general-purpose LLM equipped with web search and a multistep reasoning chain at inference time, may help explain our contrasting results.

Our study contains a number of important limitations, and the findings should be interpreted with appropriate caution and humility. First, our LLM system was constrained to reviewing text-based reports of investigations rather than the raw multimodal investigations themselves. This presents the possibility of upstream errors; however, we attempted to mitigate this by allowing cardiologists in both assisted and unassisted groups to review the raw imaging and clinical data themselves; general cardiologists noted a clinically significant omission in the text reports in fewer than 8% of cases. History and physical examination are indispensable components of real clinical practice, but they were not included in this study. This is a limitation of the applicability of our work, and future studies should consider the settings in which there is prospective interaction with these patients. However, we did offer cardiologists the ability to interact with our LLM. While our study was conducted on real patient cases, we do not consider LLMs ready for safe deployment and thus we did not deploy our LLM into live, prospective clinical care. If safety standards are met, future studies should assess the performance of LLMs in live, prospective clinical care. An additional limitation is that cardiologists were not blinded to their intervention assignment, thereby introducing a potential performance bias that may have influenced cardiologists’ subjective reports about LLM usefulness and time savings. However, our primary efficacy outcomes were protected from this bias through blinded subspecialist evaluation, and the subjective measures of user experience represent clinically meaningful assessments of technology acceptability that are relevant for real-world implementation. Further, our study relies on subspecialist preference as the primary outcome measure, which introduces inherent subjectivity despite our expert-developed evaluation rubrics. While this approach aligns with recent RCTs of AI-assisted clinical decision-making^44,45^, preference-based evaluation cannot definitively establish real-world clinical benefit. Evaluating downstream patient outcomes would require prospective studies with long-term follow-ups that are beyond the current scope.

Further limitations of our work include a biased sample of patients—patients were selected from one US center, using only English text. It is unclear how well our results will extrapolate to other non-US settings. Additionally, subspecialist evaluators were from the same institution where AMIE’s prompt engineering was developed, potentially introducing institutional bias, though this is mitigated by the use of different specialists for development versus evaluation along with minimal, held-out examples (nine cases only). Further, our dataset contained patients who were indeed referred for a suspicion of an inherited cardiac disease (correctly or incorrectly). A less biased population may be from a general cardiology clinic, where the prevalence of inherited disease is lower and with it possibly a higher chance of a false positive referral rate. However, this patient selection strategy was intentional and aligned with our research objective of evaluating whether general cardiologists could appropriately manage cases they would typically refer to subspecialty care when supported by an LLM. A similar limitation is that our patients had already completed a number of cardiac diagnostic tests. To help identify undiagnosed cases, LLMs would have to be studied in populations with less complete cardiac investigations. There was insufficient demographic or regional variation in the single-center population in our study to assess the potential for bias or health inequity, which is an important topic for AI systems in healthcare. This limitation is important, as disparities are well documented in the care of patients with inherited cardiomyopathies^46^ and should be addressed in prospective studies. A further consideration in the implementation of AMIE is the risk of automation bias, where clinicians may overly rely on AI outputs without sufficient scrutiny, potentially leading to inappropriate or unnecessary tests and management decisions. This bias has implications for patient safety, as it may result in increased healthcare costs, procedural risks and heightened patient anxiety. While AMIE demonstrated potential in enhancing cardiologists’ assessments, its use as a clinical aid requires careful oversight to prevent overreliance. For example, AMIE’s sensitive and detailed suggestions could lead to additional tests that are not clinically indicated. To mitigate these risks, clinicians interacting with AMIE must receive appropriate training to critically evaluate its outputs, ensuring they supplement clinical judgment rather than replace it.

Additionally, our research did not explore the potential benefits and risks from the perspective of patients. The early potential here demonstrates an opportunity for participatory research including the patient perspective on many potentially different workflows that could be enabled for subspecialist consultation. While AMIE’s performance was promising, our evaluation rubric highlighted notable areas for improvement, including the diagnosis and triage. The complementary and assistive utility of the technology requires extensive further study before it could be considered safe for real-world use, and there are many other considerations beyond the scope of this work, including regulatory and equity research and validation in a wider range of clinical environments.

In conclusion, AMIE, a research LLM-based AI system, can improve general cardiologists’ assessments of complex cardiac patients. Assistance from AMIE led general cardiologists to have significantly fewer errors, faster assessments, lower rates of erroneous extra content and equivalent clinical reasoning.

## Methods

AMIE is an experimental LLM-based medical AI system^9^. In this study, AMIE was built on top of Gemini 2.0 Flash without any additional domain-specific fine-tuning. Instead, AMIE used a multistep inference procedure involving web search and self-critique to adapt it to this subspecialist domain. Further details on the inference procedure are provided in Supplementary Section 1.

To assess AMIE’s ability, we developed a blinded fully counterbalanced RCT, as described in Fig. 1. The study comprised several phases: (1) recruitment and de-identification of clinical data from a subspecialized inherited cardiovascular center, (2) completion of an assessment of real clinical cases (randomized to with or without AMIE assistance) by general cardiologists and (3) subspecialist evaluation and analysis of clinical assessments who were blinded to the source of each assessment.

For the RCT, the general cardiologists were tasked with interpreting clinical text and raw clinical data from real-world patients, including ECGs, rest and stress TTEs, CMRs, ambulatory Holter monitors and CPXs. The general cardiologists were randomized to complete this assessment with assistance from AMIE or without. Assistance consisted of access to AMIE’s comprehensive clinical assessment report and the ability for general cardiologists to conversationally interact with AMIE directly in a web interface. The user interfaces are shown in Extended Data Fig. 1.

The data for this study were obtained from patients referred to SCICD, encompassing patients with both suspected and confirmed inherited cardiovascular diseases and general cardiology patients. To facilitate scientific progress and reproducibility of our results, we have made all data publicly available (see the ‘Data availability’ section). Our RCT followed the CONSORT RCT guidelines and is registered at ClinicalTrials.gov (NCT06935253).

### Clinical data

SCICD is one of the world’s largest centers focused on inherited cardiovascular disease. Patients with suspected genetic cardiovascular disease are referred for diagnosis and management. Referrals typically come from general cardiologists. The patient population largely contains patients with suspected inherited cardiovascular disease; however, the clinic also sees general cardiology patients. All physicians at SCICD have received subspecialty training in genetic cardiac disease.

The assessment of patients involves a review of the patient’s history and tests such as CMRs, rest and stress TTEs, CPXs, ECGs, ambulatory Holter monitors and genetic testing. For each test, a text report outlining in-depth and summarized test results is produced. Text results from these investigations were available and used for all patients included in this study. Further, general cardiologists had full access to the raw clinical data (for example, echocardiogram images). We utilized 107 real-world patient cases in the test set for the RCT after first exposing the model to nine different patient cases for model refinement. No cases used in the model refinement were used for model testing.

### Study design and evaluation

#### Cardiologist and AMIE assessment

For our RCT, we exposed general cardiologists to text and raw data from 107 consecutive, real-world patients. General cardiologists were selected from a pool of nine Stanford general cardiologists, with two general cardiologists assessing each patient. The test population consisted of patients suspected or confirmed to have inherited cardiovascular disease as well as a mixture of patients without genetic cardiovascular disease. The data consisted of physician text reports and the raw data for CMRs, rest and stress TTEs, CPXs, ECGs and ambulatory Holter monitors. General cardiologists were tasked with completing the same standardized assessment form shown in Extended Data Fig. 2. One of the two general cardiologists was randomized to complete the assessment with the assistance of AMIE. Assistance consisted of access to AMIE’s completed assessment form and a conversational web interface where the general cardiologist could interact with AMIE.

The assessment form (Extended Data Fig. 2) prompted general cardiologists to provide their assessment across a range of domains, including triage, diagnosis and management of these patients with potential inherited cardiovascular disease. The general cardiologists were asked to provide an overall impression of the patient’s case and answer a consult question regarding the likelihood of a genetic cardiac disease. The Triage Assessment section prompted the general cardiologists to determine the necessity of referral to a specialist center. In the Diagnosis section, they were asked to list their most likely diagnosis along with any additional information they would need to ask the patient or gather from tests. In the Management section, they were asked to describe their management plan and any additional information they would need to guide their management.

Lastly, the general cardiologists were asked whether the raw imaging contained any findings that were missing in the text reports, and if so, to comment on whether this missed information was clinically significant or insignificant. If AMIE assistance was provided, the general cardiologists were asked whether the AI use was helpful, made them more confident, saved them time and had hallucinations or omissions. They were encouraged to leave free-text comments regarding their use of AI and any hallucinations or omissions.

#### Subspecialist evaluation

The general cardiologists’ assessments were evaluated by one of three SCICD subspeciality cardiologists who evaluated pairs of general cardiologist assessments on the same patient (one with and one without assistance from AMIE). The subspecialists were blinded to the source of each assessment, and the assessments were provided in a randomized order. Subspecialists completed two types of evaluation per case: direct A/B preference comparisons between the two responses and individual assessments per response, as described in Methods, ‘Development of an evaluation rubric for subspecialist case interpretation’.

#### Development of an evaluation rubric for subspecialist case interpretation

We developed rubrics for subspecialists to evaluate the responses under two conditions: (1) a direct A/B comparison of general cardiologist’s responses with and without AMIE assistance both overall and across each domain, and (2) an individual evaluation of each general cardiologist’s response.

For the direct A/B comparison (see top portion of Extended Data Fig. 3), the domains of the evaluation rubric mirrored the clinical assessment form completed by general cardiologists. For each domain, the subspecialist evaluators indicated a direct preference between the two assessments, one completed independently by a general cardiologist and one completed by a general cardiologist with assistance from AMIE. Evaluations were completely blinded. We designed this preference comparison with a third option for a tie to facilitate greater potential discrimination in performance between the general cardiologists as compared with Likert scales, while also allowing equivalence to be expressed.

To investigate more nuanced qualities of each response, the subspecialists also evaluated the general cardiologist’s responses individually. To do this, we developed an individual evaluation rubric (see the bottom portion of Extended Data Fig. 3). This was developed by identifying response quality themes from the existing literature^8^ and iteratively combining semi-structured feedback from LLM domain experts and cardiologist experts not involved in the evaluation or the remainder of the study design. Themes from the existing literature were shared with respective experts who then provided feedback in a semi-structured manner. Themes were narrowed and refined in response to expert feedback until thematic saturation (no new themes emerged), which in our case led to five major themes. Instructions for evaluators were written and piloted using the nine development cases. These cases were piloted and used as worked examples in interactive feedback sessions with expert evaluators. Feedback on instructions and the evaluation rubric was sought from experts and changes implemented if concordance from experts was present. The above approach led to iterative improvement of our individual evaluation rubric, spanning five crucial domains of LLM evaluation: (1) errors, (2) addition, (3) omission, (4) reasoning and intelligence and (5) bias. Subspecialist experts were asked to answer “Yes” or “No” to direct questions spanning these five domains and then given free-text responses to quantify and explain their selections.

#### Statistical analysis

To test the hypothesis that the assisted condition (cardiologist + AMIE) outperformed the unassisted condition (cardiologist alone), we employed several statistical analyses. For subspecialist preference ratings (‘Direct preference: general cardiologists with and without AMIE’), we used two-proportion *z*-tests to compare the selection frequencies of cardiologist + AMIE versus cardiologist alone for each criterion. To analyze individual criteria (‘Individual assessment of general cardiologists’ responses with and without AMIE’), McNemar’s tests were performed on 2 × 2 contingency tables of paired “Yes”/“No” responses from both conditions. Error bars in Fig. 3 represent 95% confidence intervals derived from bootstrapping (*n* = 10,000).

#### Ethics approval

The clinical subspecialist evaluator component of this research involved the participation of physicians. This study adhered to the principles outlined in the Declaration of Helsinki. Informed consent was obtained from each physician before their participation. This study used only retrospective, de-identified data that fell outside the scope of institutional review board oversight.

### Reporting summary

Further information on research design is available in the Nature Portfolio Reporting Summary linked to this article.

## Online content

Any methods, additional references, Nature Portfolio reporting summaries, source data, extended data, supplementary information, acknowledgements, peer review information, details of author contributions and competing interests, and statements of data and code availability are available at 10.1038/s41591-025-04190-9.

## Supplementary information

Supplementary Information Supplementary Figs. 1–7, Tables 1–9 and Discussion Sections 1–5.

Reporting Summary

## Data Availability

Data consists of clinical test text data (ECGs, CMRs, rest and stress TTEs, ambulatory Holter monitors, CPXs). All data are open-source and are available at https://redivis.com/datasets/1z3x-2354972da?v=next. Data are licensed under open-source license CC 4.0.

## Code availability

AMIE was built on top of Gemini 2.0 Flash, which is publicly available for use, without any additional fine-tuning. The inference process used to generate assessments is detailed in Supplementary Section 1.2, while the prompt used in the conversational interface is presented in Supplementary Section 1. All analyses were conducted using Python v.2.7.18 (https://www.python.org/).
