# Supplementary material for: A large language model for complex cardiology care
Source: Nat Med. 2026 Feb 6;32(2):616–23. doi: 10.1038/s41591-025-04190-9 (PMC12920087; doi:10.1038/s41591-025-04190-9)
Supplement: Supplementary file 1 — Supplementary Figs. 1–7, Tables 1–9 and Discussion Sections 1–5. [file 41591_2025_4190_MOESM1_ESM.pdf]

nature medicine

Supplementary information https://doi.org/10.1038/s41591-025-04190-9

A large language model for complex cardiology care

In the format provided by the authors and unedited

Supplementary Information

In the following sections, we provide additional details for the RCT:

- Details on the development of AMIE and the inference procedure (Section SI.1)
- Distribution of responses to the AI use questions for each general cardiologist (Section SI.2)
- Examples of an AMIE conversations with general cardiologists (Section SI.3)
- Automated analysis of general cardiologists' comments (Section SI.4)
- Automated analysis of subspecialist cardiologists' comments (Section SI.5)

SI.1 AMIE system details

SI.1.1 Model development

The AMIE system in this study is built upon Gemini 2.0 Flash. At inference time, AMIE utilizes a chain-of-reasoning strategy adaptable to various medical tasks, enabling the integration of automated feedback mechanisms and tool use to refine its response. In this work, we do not perform any additional training to modify AMIE. Instead, we only modify the prompting and the inference strategy to adapt AMIE to this new domain.

SI.1.2 Domain adaptation of AMIE

Adaptation of AMIE to this subspecialist domain required clinical data from just nine patients; this small number of cases was sufficient because we were not performing any model training and instead focused on modifying the inference strategy. Five cases, chosen at random, were used to design an optimal approach to model prompting through iterative review and expert feedback. Several prompting strategies were tested: zero vs. few-shot prompting, with vs. without search retrieval augmentation, providing an AMIE generated summary of the cardiac testing information vs. providing raw cardiac testing information, and using the original response vs. allowing the model to self-critique and revise its answer. We had a subspecialist cardiologist review the responses on these 5 validation cases and select their preferred set of responses. Ultimately, the specialist preferred the "few-shot with self-critique and search retrieval augmentation" method. This method worked as follows:

1. **Few-shot draft:** Using four case examples and "gold-standard" assessments from a subspecialist cardiologist, AMIE is asked to draft an assessment for a new case.
2. **Web-search:** AMIE generates relevant search queries (i.e., "What is the MYH7 gene", "HCM genetic testing implications for treatment") and summarizes the search results.
3. **Self-critique:** AMIE critiques its original draft assessment using these web-search results, listing positive and negative aspects of its draft.
4. **Revision:** AMIE creates a revised version of its assessment by factoring in its self-critique to produce a final assessment.

The few-shot exemplars used were four additional patient cases for which a specialist cardiologist provided a "gold-standard" case assessment. Note that none of the nine patient cases used as either few-shot exemplars or for validating inference strategies were part of the test set.

SI.1.3 Conversation with AMIE

AMIE's assessment was produced via the method described in Section SI.1.2. In the conversational interface (see Figure ED.1a), AMIE was instructed to respond helpfully to the cardiologist using the prompt in Figure SI.1.

AMIE Conversation Prompt

You are an expert medical AI system, AMIE, chatting with a general cardiologist about a particular patient's case. You have already assessed the case and have come up with a thorough assessment of the patient's condition. The cardiologist has seen your assessment. Now they would like to chat with you about the case and related clinical details.
Respond to the cardiologist's questions, and intelligently speak about the patient's case and your assessment. You should exhibit the expertise of a highly experienced subspecialty cardiologist. Think about your response carefully before responding. Below are details of the case and your assessment: {Scenario} {AMIE assessment}

Figure SI.1 | Prompt to AMIE for responding to general cardiologists.

SI.2 Questionnaire responses per cardiologist

Table SI.1 | Questionnaire responses per cardiologist. Proportion of answer choices from each cardiologist for the five questions on AI use and the question on raw imaging artifacts from Figure ED.2. F represents Female and M represents Male.

**Cardiologist**	**C1**	**C2**	**C3**	**C4**	**C5**	**C6**	**C7**	**C8**	**C9**
**Sex**	F	F	M	F	M	F	M	F	M
**Did the AI help your assessment?**									
Yes, definitely	0.19	0.12	0.11	0.2	0.0	0.0	0.71	0.0	0.0
Yes, probably	0.38	0.41	0.32	0.4	0.75	0.8	0.29	0.38	0.1
Neither yes nor no	0.19	0.41	0.53	0.2	0.12	0.0	0.0	0.38	0.8
No, probably not	0.12	0.06	0.05	0.2	0.0	0.1	0.0	0.25	0.1
No, definitely not	0.12	0.0	0.0	0.0	0.12	0.1	0.0	0.0	0.0
**Did the AI make you more confident?**									
Yes, definitely	0.19	0.0	0.21	0.0	0.0	0.0	0.64	0.12	0.0
Yes, probably	0.31	0.47	0.47	0.6	0.12	0.3	0.36	0.5	0.1
Neither yes nor no	0.25	0.53	0.21	0.2	0.75	0.3	0.0	0.12	0.7
No, probably not	0.12	0.0	0.11	0.2	0.12	0.3	0.0	0.25	0.2
No, definitely not	0.12	0.0	0.0	0.0	0.0	0.1	0.0	0.0	0.0
**Did the AI save you time?**									
Saved me more than 50% of my time	0.12	0.41	0.11	0.0	0.0	0.0	0.93	0.12	0.0
Saved me more than 10% of my time	0.5	0.41	0.37	0.6	0.0	0.1	0.07	0.25	0.0
Not sure	0.25	0.18	0.32	0.4	0.88	0.5	0.0	0.38	0.3
Delayed me by more than 10%	0.12	0.0	0.21	0.0	0.12	0.2	0.0	0.12	0.7
Delayed me by more than 50%	0.0	0.0	0.0	0.0	0.0	0.2	0.0	0.12	0.0
**Did the AI have any hallucinations?**									
No	0.94	0.94	0.95	0.8	0.88	1.0	1.0	0.88	0.7
At least one hallucination but not clinically significant	0.06	0.0	0.0	0.2	0.0	0.0	0.0	0.0	0.0
At least one clinically significant hallucination	0.0	0.06	0.05	0.0	0.12	0.0	0.0	0.12	0.3
**Did the AI miss anything?**									
No	1.0	0.94	1.0	1.0	1.0	0.8	1.0	0.88	0.7
At least one clinically significant omission	0.0	0.06	0.0	0.0	0.0	0.2	0.0	0.12	0.3
**Did you notice any clinically important findings on the raw imaging that was not mentioned in the text-only report?**									
No	0.53	0.65	0.68	0.5	1.0	0.85	0.86	0.81	0.85
At least one omission but not clinically significant	0.41	0.18	0.26	0.3	0.0	0.05	0.07	0.19	0.1
At least one clinically significant	0.06	0.18	0.05	0.2	0.0	0.1	0.07	0.0	0.05

SI.3 Examples of AMIE interactions

SI.3.1 Example of AMIE conversation 1

Table SI.2 | Example conversation 1

Speaker	Response
**Cardiologist**	What is the significant for the Vo2 results?
**AMIE**	The VO2 results are significant for several reasons:<br>1. **Reduced Aerobic Capacity:** The patient's VO2max of 20.7 ml/kg/min, which is 87% of predicted, indicates a mild impairment in aerobic capacity. This means their ability to utilize oxygen during exercise is reduced compared to what is expected for their age and sex. This is consistent with the patient's symptoms of fatigue and exercise intolerance.<br>2. **Exercise Limitation:** The exercise test was terminated due to general fatigue, which is a subjective measure, but it is consistent with the objective finding of reduced VO2 max. This suggests that the patient's exercise capacity is limited by their cardiovascular or respiratory system.<br>3. **Abnormal Exercise ECG Response:** The presence of several isolated PVCs during exercise is abnormal and suggests an increased risk of ventricular arrhythmias. This is particularly concerning in the context of the patient's mildly reduced LVEF and high PVC burden on the Zio patch.<br>4. **Elevated VE/VCO2 Slope:** The VE/VCO2 slope of 38.0 (and 35.0 at the anaerobic threshold) is significantly elevated. This indicates inefficient ventilation during exercise and is an adverse prognostic marker, particularly in heart failure patients. It suggests that the patient is ventilating excessively for the amount of CO2 being produced, which can be due to a number of factors, including pulmonary congestion, increased dead space ventilation, and impaired cardiac output.<br>5. **Oscillatory Breathing:** The presence of oscillatory breathing is also abnormal and can be a sign of underlying heart failure or other cardiopulmonary issues. It suggests instability in the respiratory control system.<br>6. **Stable VO2max:** The fact that the VO2max is similar to the previous test in 2019 suggests that the patient's exercise capacity has not significantly worsened over time. However, the elevated VE/VCO2 slope and the presence of PVCs during exercise are new findings that warrant further investigation.<br>In summary, the VO2 results indicate that the patient has a reduced aerobic capacity, abnormal exercise ECG response, inefficient ventilation, and oscillatory breathing, all of which are concerning and support the suspicion of an underlying cardiomyopathy. The elevated VE/VCO2 slope is particularly important as it is an adverse prognostic marker in heart failure.
**Cardiologist**	What findings of the echo are most suspicious for genetic cardiomyopathy:

Speaker	Response
**AMIE**	Several findings on the echocardiogram are suspicious for a genetic cardiomyopathy, particularly dilated cardiomyopathy (DCM):<br>1. **Mildly Reduced Left Ventricular Systolic Function:** The LVEF of 53% on the resting echo and 49% on the stress echo, while only mildly reduced, is a key finding. In the absence of other clear causes (like significant coronary artery disease), this degree of systolic dysfunction is often seen in early or mild DCM.<br>2. **Normal LV Size with Mildly Reduced Systolic Function:** The combination of normal LV size with reduced systolic function is more suggestive of DCM than other cardiomyopathies like hypertrophic cardiomyopathy (HCM), where you would expect to see increased wall thickness.<br>3. **Mildly Dilated Left Atrium:** The mildly dilated left atrium (LA volume index of 36.4 ml/m2) suggests chronic elevation of left ventricular filling pressures, which is consistent with diastolic dysfunction and can be seen in DCM.<br>4. **Abnormal Diastolic Function:** The resting echo notes abnormal diastolic function with borderline elevated LV filling pressures. This is a common finding in DCM.<br>5. **Mildly Thickened Mitral and Aortic Valve Leaflets:** While not specific, mild thickening of the mitral and aortic valve leaflets can be seen in some cardiomyopathies, including DCM.<br>6. **Reduced Global Longitudinal Strain (GLS):** The reduced LV GLS of -18.7% at rest and -16.5% with stress is a significant finding. GLS is a sensitive marker of myocardial function, and a reduction suggests impaired contractility, often seen in DCM.<br>7. **Absence of Stress-Induced Wall Motion Abnormality:** The stress echo showed mild diffuse hypokinesis without stress-induced wall motion abnormality (WMA). This makes ischemic cardiomyopathy less likely.<br>8. **Normal RV Size and Function:** While not directly suggestive of DCM, the normal RV size and function help to rule out other conditions that might affect both ventricles.<br>In summary, the combination of mildly reduced LVEF, normal LV size, diastolic dysfunction, mildly dilated LA, reduced GLS, and the absence of stress-induced WMA are all findings that raise suspicion for a genetic cardiomyopathy, most likely DCM.
**Cardiologist**	Why would this not be ischemic in etiology?
**AMIE**	There are several reasons why an ischemic etiology is less likely:<br>1. **Absence of Typical Anginal Symptoms:** The patient's history does not mention typical anginal symptoms such as chest pain or pressure brought on by exertion.<br>2. **Normal Stress Echo (No Stress-Induced WMA):** The stress echo showed no stress-induced wall motion abnormalities (WMA), a hallmark of ischemic heart disease.<br>3. **Diffuse Hypokinesis:** The observed mild diffuse hypokinesis is more consistent with a global cardiomyopathy like DCM, rather than regional ischemia.<br>4. **Normal Coronary Anatomy (Presumed):** The absence of a history suggestive of coronary artery disease (CAD) makes significant CAD less likely.<br>5. **Global Longitudinal Strain (GLS) Reduction:** The reduction in GLS is a global finding, more consistent with a cardiomyopathy than regional ischemia.<br>6. **Other Findings More Consistent with DCM:** Mildly reduced LVEF, diastolic dysfunction, a mildly dilated LA, and significant ventricular arrhythmias are all more consistent with DCM.<br>7. **Age:** The patient's age (57) is not particularly high for ischemic heart disease, especially without other risk factors.<br>8. **Exercise Test Findings:** The elevated VE/VCO2 slope and oscillatory breathing are more consistent with heart failure and cardiomyopathy than with ischemia.<br>In summary, the clinical picture, stress echo findings, and other test results point towards a non-ischemic cardiomyopathy as the more likely diagnosis.
**Cardiologist**	What other questions should I ask the patient?

<table>
<tr><th>Speaker</th><th>Response</th></tr>
<tr><td>AMIE</td><td>Here are additional questions, categorized for clarity:
1. Detailed Symptom History:
• Specific Palpitation Characteristics: Can you describe your palpitations (e.g., fast, slow, regular, irregular, pounding, fluttering)? How long do they usually last? What triggers them (e.g., exercise, stress, caffeine)? Do they occur with other symptoms like dizziness or shortness of breath?
• Detailed Exercise Intolerance: How has your exercise tolerance changed over time? What specific activities are you unable to do now that you could do before? Do you experience shortness of breath, chest discomfort, or fatigue with exertion?
• Syncope/Pre-Syncope: Have you ever experienced any fainting or near-fainting episodes? If so, what were you doing? Did you have any warning signs?
• Other Symptoms: Do you experience any swelling in your legs or ankles, difficulty breathing when lying flat, or unexplained fatigue or weakness?
2. Family History (More Specific):
• Sudden Unexplained Deaths: Have there been any sudden, unexpected deaths in your family, especially at a young age (under 50)? Were any attributed to unexplained causes like drowning or car accidents?
• Specific Heart Conditions: Has anyone in your family been diagnosed with cardiomyopathy, heart failure, had a pacemaker/defibrillator implanted, had a heart transplant, a stroke/TIA, or atrial fibrillation?
• Family Structure: Are your parents related (consanguinity)? Are you aware of any family members who have had genetic testing for heart conditions?
3. Lifestyle and Medical History:
• Alcohol and Drug Use: Can you describe your alcohol consumption in detail? Have you ever had a period of heavy alcohol use? Have you ever used recreational or performance-enhancing drugs?
• Other Medical History: Can you provide a complete list of all medications, including over-the-counter and supplements? Have you ever been diagnosed with other conditions (e.g., diabetes, hypertension, thyroid problems, autoimmune diseases)?
4. Psychosocial and Test-Related Factors:
• Emotional Well-being and Support: How are you feeling emotionally about your health? Do you have a good support system?
• Event Correlation: Can you describe what you were doing during the palpitations recorded on the Zio patch? How did you feel during the exercise test?</td></tr>
<tr><td>Cardiologist</td><td>What genetic mutations are most likely for these constellation of findings?</td></tr>
<tr><td>AMIE</td><td>Given the findings, the most likely genetic mutations would be those associated with dilated cardiomyopathy (DCM). Key genes include:
• TTN (Titin): This is the most common gene in DCM (up to 25% of cases). Mutations often present with reduced LVEF, arrhythmias, and progressive heart failure, similar to this patient.
• LMNA (Lamin A/C): Associated with DCM and conduction system disease/arrhythmias. Can also be associated with skeletal muscle disease.
• MYH7 (Myosin Heavy Chain 7): While more common in hypertrophic cardiomyopathy (HCM), some mutations cause DCM.
• Other Genes: Other possibilities include DMD (Dystrophin), DES (Desmin), SGC (Sarcoglycans), FLNC (Filamin C), RBM20, PLN (Phospholamban), and BAG3, all of which are linked to DCM and sometimes arrhythmias or myopathy.
Recommendation: A comprehensive cardiomyopathy genetic panel is recommended, with referral to a genetic counselor.</td></tr>
<tr><td>Cardiologist</td><td>Are there other tests I should pursue beyond genetic testing?</td></tr>
</table>

Speaker	Response
**AMIE**	Yes, absolutely. The following tests should be pursued to get a comprehensive picture:<br>• **High Priority Tests:**<br>– **Cardiac MRI (CMR) with LGE:** Critical for detailed assessment of cardiac structure, function, and tissue characterization (e.g., fibrosis, scarring, or infiltration) to help refine the diagnosis.<br>– **Extended ECG Monitoring (Holter/Zio):** To quantify the burden and complexity of ventricular arrhythmias and assess the risk of sudden cardiac death.<br>– **BNP or NT-proBNP:** To assess the severity of heart failure and monitor response to therapy.<br>• **Consider Based on Clinical Picture:**<br>– **Electrophysiology Study (EPS):** To assess the inducibility of sustained ventricular tachycardia (VT) if arrhythmias are a major concern.<br>– **Endomyocardial Biopsy:** If CMR suggests an infiltrative process (e.g., sarcoidosis) or the diagnosis remains unclear.<br>– **Right Heart Catheterization:** To directly measure cardiac pressures and output if there is suspicion of advanced heart failure or pulmonary hypertension.<br>– **Repeat Cardiopulmonary Exercise Testing (CPET):** To track exercise capacity and guide activity recommendations over time.
**Cardiologist**	Please summarize the abnormal findings in this patient.
**AMIE**	Here is a summary of the patient's key abnormal findings:<br>• **Cardiac Dysfunction:** Mildly reduced LVEF (53% at rest, 49% with stress), abnormal diastolic function with borderline elevated filling pressures, and reduced global longitudinal strain (GLS) of -18.7%.<br>• **Arrhythmias:** Significant PVC burden (6.0%), one episode of non-sustained ventricular tachycardia (NSVT), and several isolated PVCs during exercise.<br>• **Exercise Intolerance:** Mildly impaired aerobic capacity (VO2max 87% predicted) with test termination due to fatigue, inefficient ventilation (elevated VE/VCO2 slope of 38.0), and oscillatory breathing.<br>• **Structural and ECG Findings:** Mildly dilated left atrium, mildly thickened valve leaflets, and a borderline ECG with low precordial voltage and T-wave abnormalities.<br>**Overall Impression:** The constellation of findings strongly suggests an underlying non-ischemic cardiomyopathy, most likely dilated cardiomyopathy (DCM), with a high likelihood of a genetic etiology. The combination of cardiac dysfunction, significant arrhythmias, and impaired exercise capacity is highly concerning and warrants comprehensive investigation.

SI.3.2 Example of AMIE conversation 2

Table SI.3 | Example conversation 2

<table>
<tr><th>Speaker</th><th>Response</th></tr>
<tr><td>Cardiologist</td><td>What are the overlap between amyloid and ATTR?</td></tr>
<tr><td>AMIE</td><td>Great question. Let’s clarify the relationship between amyloidosis and ATTR amyloidosis:
Amyloidosis is the overarching term for a group of diseases characterized by the abnormal deposition of misfolded proteins (amyloid fibrils) in various tissues and organs. These deposits disrupt normal tissue function. There are many types of amyloidosis, classified by the specific protein that forms the amyloid fibrils.
ATTR amyloidosis (Transthyretin amyloidosis) is a specific type of amyloidosis where the amyloid fibrils are formed from the protein transthyretin (TTR). TTR is a protein primarily produced in the liver that transports thyroid hormone and retinol.
Here’s a breakdown of the overlap:
• ATTR is a subtype of amyloidosis: All cases of ATTR amyloidosis are, by definition, also cases of amyloidosis. However, not all cases of amyloidosis are ATTR amyloidosis. Other types include AL amyloidosis (light chain), AA amyloidosis, etc.
• Shared Pathophysiology: Both involve the deposition of amyloid fibrils, leading to organ dysfunction. In the heart, this results in restrictive cardiomyopathy, diastolic dysfunction, and conduction abnormalities.
• Clinical Overlap: Both can present with similar symptoms, including heart failure, peripheral neuropathy, carpal tunnel syndrome, and autonomic dysfunction.
• Diagnostic Overlap: Both require imaging (e.g., cardiac MRI, echocardiography) and tissue biopsy (if needed) for diagnosis.
Key Differences:
• Causative Protein: The key difference is the specific protein involved. In ATTR, it’s TTR; in AL, it’s immunoglobulin light chains, etc.
• Treatment: Treatment strategies are different for each type of amyloidosis. ATTR amyloidosis has specific therapies (Tafamidis, Patisiran, Inotersen) that target TTR, while AL amyloidosis requires chemotherapy to reduce the production of light chains.
• Genetic Basis: ATTR amyloidosis can be hereditary (variant ATTR or ATTRm) or non-hereditary (wild-type ATTR or ATTRwt). AL amyloidosis is not hereditary.
In summary: Think of "amyloidosis" as the broad category, and "ATTR amyloidosis" as a specific type within that category. All ATTR amyloidosis is amyloidosis, but not all amyloidosis is ATTR. The distinction is crucial because it dictates the appropriate diagnostic workup and treatment.
In this patient, the clinical picture (age, severe hypertrophy, LGE pattern, elevated T1/T2, conduction disease) strongly suggests amyloidosis, and given the age, ATTR is the most likely subtype. However, it is essential to rule out AL amyloidosis first with serum and urine studies.
Does this help clarify the overlap between amyloidosis and ATTR amyloidosis?</td></tr>
<tr><td>Cardiologist</td><td>What features overlap and are distinct for ATTR amyloid and HCM?</td></tr>
</table>

Speaker	Response
**AMIE**	That's an excellent and clinically relevant question. Differentiating between ATTR amyloidosis and hypertrophic cardiomyopathy (HCM) can be challenging as they share overlapping features but also have distinct characteristics. Here's a breakdown:<br>**Overlapping Features:**<br>• **Left Ventricular Hypertrophy (LVH):** Both conditions can cause significant thickening of the left ventricular walls, often the initial finding that raises suspicion for either diagnosis.<br>• **Diastolic Dysfunction:** Both commonly lead to impaired relaxation and filling of the left ventricle, resulting in diastolic dysfunction and heart failure symptoms.<br>• **Elevated LV Mass:** Both can result in an increased left ventricular mass on imaging studies.<br>• **Conduction Abnormalities and Arrhythmias:** Both can cause conduction system disease (e.g., bundle branch blocks) and predispose patients to atrial and ventricular arrhythmias.<br>• **Heart Failure Symptoms:** Both can present with similar heart failure symptoms, such as dyspnea, fatigue, and edema.<br>**Distinct Features Favoring ATTR Amyloidosis:**<br>• **Pattern of LVH: ATTR:** Typically causes a more diffuse and concentric pattern of LVH, often with increased thickness of the right ventricle and atria. **HCM:** More commonly causes asymmetric septal hypertrophy, although other patterns can occur.<br>• **Late Gadolinium Enhancement (LGE) on Cardiac MRI: ATTR:** Characteristically shows a patchy, subendocardial, mesomyocardial, and sometimes transmural pattern of LGE, often extensive and present in the right ventricle and atria. **HCM:** Typically shows LGE in the areas of maximal hypertrophy, often in the mid-myocardium, and is usually less extensive.<br>• **Native T1 and T2 Mapping on Cardiac MRI: ATTR:** Shows significantly elevated native T1 and T2 values due to amyloid infiltration, a key differentiating feature. **HCM:** Usually has normal or only mildly elevated T1 and T2 values.<br>• **Echocardiographic Findings: ATTR:** May show a "granular sparkling" appearance of the myocardium. The LVEF may be preserved initially but often decreases as the disease progresses. **HCM:** May show systolic anterior motion (SAM) of the mitral valve and LV outflow tract obstruction (LVOTO), which is not typical in ATTR.<br>• **ECG Findings: ATTR:** Often shows low QRS voltage, which is not typical in HCM. **HCM:** May show deep Q waves and increased QRS voltage.<br>• **Extracardiac Manifestations: ATTR:** Often associated with extracardiac manifestations such as carpal tunnel syndrome, peripheral neuropathy, and spinal stenosis. **HCM:** Typically does not have these extracardiac manifestations.<br>• **Age of Onset: ATTR:** More common in older adults, particularly wild-type ATTR. **HCM:** Can present at any age, including childhood and adolescence.<br>**In summary:** While both ATTR amyloidosis and HCM can cause LVH and diastolic dysfunction, the *pattern* of hypertrophy, the *extent and pattern* of LGE, the *degree of elevation* in T1/T2 values, the presence of *extracardiac manifestations*, and the *age of onset* are key features that help distinguish between the two. In this patient, the extensive and atypical LGE, significantly elevated T1/T2 values, and age strongly favor ATTR amyloidosis over HCM.<br>Does this help clarify the overlapping and distinct features of ATTR amyloidosis and HCM?

SI.3.3 Example of AMIE conversation 3

Table SI.4 | Example conversation 3

Speaker	Response
**Cardiologist**	what is the association between HCM and bicuspid aortic valve
**AMIE**	That's a great question. The association between hypertrophic cardiomyopathy (HCM) and bicuspid aortic valve (BAV) is not fully understood, but it's increasingly recognized as more than just a coincidental finding. Here's a breakdown of the current understanding:<br><br>**1. Shared Genetic Pathways:**<br>**Overlapping Genetic Predisposition:** Both HCM and BAV have strong genetic components. While HCM is primarily linked to mutations in sarcomere protein genes, and BAV is associated with genes involved in valve development and extracellular matrix, there's evidence of overlapping genetic pathways. Some genes, like those involved in TGF-β signaling, have been implicated in both conditions.<br>**Syndromic Associations:** Certain genetic syndromes, such as Noonan syndrome and related RASopathies, can present with both HCM and BAV. These syndromes involve mutations in genes that affect multiple developmental pathways, leading to a variety of cardiac and extracardiac manifestations.<br>**2. Developmental Links:**<br>**Embryonic Development:** Both the aortic valve and the ventricular myocardium develop during similar stages of embryogenesis. Disruptions in these early developmental processes could potentially lead to the co-occurrence of BAV and HCM.<br>**Hemodynamic Stress:** It's hypothesized that the abnormal hemodynamics associated with a BAV (e.g., altered flow patterns, increased shear stress) could contribute to the development or progression of LV hypertrophy in some individuals. However, this is not a universal finding, and many patients with BAV do not develop HCM.<br>**3. Clinical Implications:**<br>**Increased Risk of Aortopathy:** Patients with both HCM and BAV may have an increased risk of developing aortic dilation or aneurysm, compared to those with either condition alone. This is likely due to the combined effects of altered hemodynamics and underlying genetic predispositions.<br>**More Complex Management:** The presence of both conditions can complicate management decisions. For example, the presence of aortic stenosis from the BAV may make it more difficult to assess the severity of LVOT obstruction in HCM.<br>**Family Screening:** The co-occurrence of HCM and BAV in a patient should prompt a thorough family history and consideration of genetic testing, as both conditions can be inherited.<br>**4. Research and Ongoing Questions:**<br>**Phenotypic Variability:** The clinical presentation of patients with both HCM and BAV can be highly variable, ranging from asymptomatic to severe disease. This variability is likely influenced by the specific genetic mutations involved, as well as environmental factors.<br>**Need for Further Studies:** More research is needed to fully elucidate the genetic and developmental mechanisms underlying the association between HCM and BAV. This will help to improve risk stratification, diagnosis, and management of these patients.<br><br>**In summary:**<br>The association between HCM and BAV is likely multifactorial, involving shared genetic pathways, developmental links, and hemodynamic interactions. The presence of both conditions in a patient should raise suspicion for an underlying genetic predisposition and prompt a thorough evaluation, including genetic testing and family screening. It's important to recognize that this is an area of ongoing research, and our understanding of this association is continuing to evolve.<br>Do you have any other questions about this?

SI.4 Automated analysis of general cardiologists' comments

General cardiologists were encouraged to leave comments regarding their use of AI in the study. A total of 41 out of 107 cases had a comment left on the use of AI in the study. These are distinct from the specific comments left regarding hallucinations or omissions from AMIE (described in Section 2.4.1). We used Gemini 2.5 Pro to analyze the AI-use comments, using the prompt in Figure SI.2.

Prompt for analysis of general cardiologists' comments

The following are a set of comments general cardiologists left regarding the use of AMIE, an medical AI assistant, to assist in assessing a total of N=107 cases. A comment may describe both positive and negative feedback regarding the use of AMIE. Create a taxonomy of up to 10 types of errors seen and up to 10 types of benefits seen, and generate a report detailing: a) The key positive feedback about the AI use. b) The key negative feedback about the AI use. c) Counts and percentages out of the 107 cases of both positive and negative instances of each benefit/error type in a latex table.

Figure SI.2 | Prompt to Gemini 2.5 Pro for analysis of general cardiologist comments from the RCT.

The text and tables in Section SI.4.1 are the result of this prompt.

SI.4.1 AI analysis of cardiologist feedback on the use of AMIE

Gemini 2.5 Pro Analysis of General Cardiologist Comments on use of AMIE

This report provides a systematic analysis of comments from general cardiologists regarding the use of the AMIE AI assistant. The analysis is based on **41 distinct comments** provided during the assessment of a total of **N=107 clinical cases**. The feedback has been categorized into a taxonomy of observed benefits and errors to quantify the AI's performance and guide future improvements. All percentages are calculated out of the total 107 cases.

a) Key Positive Feedback about the AI Use

The most significant positive feedback centered on the AI's role as an **educational tool and knowledge resource**. Cardiologists frequently praised its ability to provide detailed information on rare diseases (e.g., DSP, PRKAG2, BMD), specific diagnostic criteria (e.g., for ICD placement), and potential disease manifestations that were not top-of-mind for the user.

Another key benefit was **efficiency and cognitive support**. The AI was valued for generating comprehensive summaries and management plans that could be used as a starting point, saving time on typing and reducing the risk of memory errors. Users appreciated the "customized check-list" of potential tests and therapies, which served as a valuable reminder.

Finally, the AI was seen as a useful **diagnostic partner**. It helped justify conclusions, increased physician confidence in challenging cases, and sometimes caught subtle findings missed by the human reviewer (e.g., chamber discrepancies between imaging modalities). In some instances, the AI's strong (though sometimes incorrect) opinion prompted cardiologists to question their own assumptions and seek more information, functioning as a catalyst for deeper critical thinking.

b) Key Negative Feedback about the AI Use

The most pervasive negative feedback was that the AI's responses were **excessively verbose and lengthy**. Cardiologists across numerous comments found the output "long-winded," "too detailed," and difficult to parse, which ironically reduced efficiency.

A critical area of concern was the AI's tendency to make **premature or overly aggressive recommendations**. It often suggested specific treatments (e.g., beta-blockers), urgent specialist referrals, or patient counseling (e.g., for an ICD) before a definitive diagnosis was established, failing to appreciate the standard stepwise clinical workflow.

Other significant errors included **factual inaccuracies (hallucinations)**, where the AI based its reasoning on findings not present in the case file (e.g., non-existent CMR or ECG findings), and an **overconfidence or dogmatic tone** in its diagnostic assertions, even when challenged with more plausible alternatives. Lastly, a number of users experienced **technical failures** where the AI was non-responsive.

c) Taxonomy, Counts, and Percentages of Benefits and Errors

The following tables detail the taxonomy of benefits and errors derived from the user comments:

Figure SI.3 | Gemini 2.5 Pro analysis of general cardiologists' comments on their use of AMIE. See Table SI.5 and Table SI.6 for the Gemini 2.5 Pro-generated tabulation of benefits/errors described in these comments.

Table SI.5 | Benefits of AI use observed by the general cardiologists. Gemini 2.5 Pro analyzed the comments left by general cardiologists and categorized the benefits described in their comments.

Benefit Type	Description	Count	Percentage
1. Knowledge Resource	Provided useful, detailed information on rare conditions, diagnostic criteria, or specific clinical findings.	5	4.7%
2. Comprehensive Checklist / Reminder	Generated a thorough list of potential tests, management steps, or differential diagnoses, acting as a cognitive aid.	4	3.7%
3. Accuracy & Agreement	The AI's assessment was accurate, appropriate, and aligned with the cardiologist's expert opinion.	3	2.8%
4. Provokes Critical Thinking	The AI's analysis, even if not perfectly correct, prompted the user to reconsider their initial assessment or seek more information.	2	1.9%
5. Interactive Dialogue / Clarification	The ability to ask clarifying questions and discuss the case with the AI was found to be helpful.	2	1.9%
6. Clinical Insight & Error Catching	Identified a subtle clinical finding or discrepancy that the user had initially missed.	1	0.9%
7. Efficiency & Time-Saving	Helped save time by generating text that could be copied, pasted, and edited, reducing manual typing.	1	0.9%
8. Justification of Rationale	Aided in articulating and justifying the reasoning behind a clinical conclusion.	1	0.9%

Table SI.6 | Issues of AI use observed by the general cardiologists. Gemini 2.5 Pro analyzed the comments left by general cardiologists and categorized the errors described in their comments.

Error Type	Description	Count	Percentage
1. Verbose / Lacks Conciseness	The AI's output was too long, overly detailed, or poorly formatted, making it difficult to use efficiently.	9	8.4%
2. Overly Aggressive / Premature Recommendations	Suggested interventions, referrals, or counseling before a diagnosis was confirmed or sufficient workup was completed.	5	4.7%
3. Overconfidence / Dogmatism	Expressed an inappropriately high degree of certainty in a diagnosis and was resistant to alternative suggestions.	5	4.7%
4. Factual Inaccuracy / Hallucination	Based its reasoning or recommendations on information that was not present in the provided case materials.	3	2.8%
5. Technical Failures	The AI tool failed to process the query or provide a response due to a backend error.	3	2.8%
6. Lack of Clinical Nuance	Failed to appreciate subtle but important clinical concepts (e.g., patchy disease) or oversimplified complex scenarios.	3	2.8%
7. Failure to Interpret Temporal Data	Incorrectly synthesized data from different time points (e.g., pre- and post-transplant) into a single diagnosis.	2	1.9%
8. Clinically Irrelevant Suggestions	Included highly unlikely differential diagnoses or tests that would not be appropriate for the clinical scenario.	1	0.9%

SI.5 Automated analysis of subspecialist cardiologists' comments

A total of 138 comments, across 7 preference categories (Overall Impression, Consult Question, Triage, Diagnosis, Diagnosis Further Questions, Diagnosis Further Tests, and Management Plan) were provided by the subspecialist cardiologists. We used Gemini 2.5 Pro to analyze these comments to interpret their reasons for preference between the AI-assisted and unassisted cardiologist responses using the prompt in Figure SI.4. Gemini's outputted report for this analysis is shown in Section SI.5.1.

Similarly, a total of 184 informative comments, across 4 of the individual evaluation categories (Clinically significant errors, extra content, missing content, correct reasoning steps) were provided by the subspecialists. We again used Gemini 2.5 Pro to analyze these comments and interpret their reasons for saying yes or no to these questions, with the prompt shown in Figure SI.5. Gemini's outputted report for this analysis is shown in Section SI.5.2.

Prompt for Analysis of Subspecialist Preferences

Subspecialist cardiologists are evaluating two responses to cardiology cases. One from a group of AI assisted cardiologists and the other from a group of unassisted cardiologists. Analyze the feedback across several dimensions, and for each dimension as well as overall, create a report summarizing the key reasons why each group was preferred. The report should be brief and include 1) Summarized overall reasons for preference for each group, and 2) For each category, a summarized reason for preference for each group.

Figure SI.4 | Prompt to Gemini 2.5 Pro for analysis of the subspecialist cardiologists' comments from the RCT for preference ratings. See resulting report in Section SI.5.1.

Prompt for Analysis of Subspecialist Individual Assessment Ratings

Subspecialist cardiologists are evaluating two responses to cardiology cases. One from a group of AI assisted cardiologists and the other from a group of unassisted cardiologists.

1. Analyze the feedback across these dimensions, and create a report summarizing the key feedback for each dimension, comparing the trends of feedback for the assisted vs unassisted cardiologists.
2. Categorize the comments for each dimension and count the instances of different error types for the unassisted and AI-assisted cardiologists in a latex table. Also produce a latex table showing illustrative examples of each error type.

Figure SI.5 | Prompt to Gemini 2.5 Pro for analysis of the subspecialist cardiologists' comments from the RCT for individual assessment ratings. See resulting report in Section SI.5.2.

SI.5.1 AI analysis of subspecialist preference comments

Gemini 2.5 Pro Analysis of Subspecialist Preference Comments

Overall Summary of Preference.
AI-Assisted Cardiologists were preferred for: Their responses were consistently more thorough, detailed, and comprehensive. They demonstrated stronger clinical reasoning by considering a broader differential diagnosis and incorporating modern diagnostic tests (e.g., genetic testing, CPX) and advanced management options (e.g., ICDs, mavacamten) into their plans.

Unassisted Cardiologists were preferred for: Their responses demonstrated grounded clinical accuracy and prudence. They excelled in cases where they correctly identified a critical piece of patient history missed by the AI (e.g., adriamycin exposure) or avoided suggesting treatments that were not yet indicated, showing sound clinical judgment.

Figure SI.6 | Gemini 2.5 Pro analysis of subspecialists' preference comments. Analysis per evaluation criteria is presented in Table SI.7.

Table SI.7 | Gemini 2.5 Pro's Analysis of subspecialists' comments: Preference rationale per preference axis.

Axis	Reason for AI-Assisted Preference	Reason for Unassisted Preference
**Overall Impression**	Provided more thorough, well-reasoned, and comprehensive answers that often included advanced therapies and testing.	Showed greater accuracy in specific situations by correctly incorporating key patient history or avoiding over-interpretation of data.
**Consult Question**	Offered stronger clinical reasoning by integrating multiple data points and considering a wider range of possibilities (e.g., bicuspid valve).	Remained more focused and accurate in cases where the AI missed a critical historical detail.
**Triage**	*No clear preference noted for this group.*	Correctly incorporated critical patient history (e.g., chemotherapy) into the decision-making process.
**Diagnosis**	Included more specific, high-probability diagnoses (e.g., HCM) and relevant associated conditions in the differential.	Provided a broader and more reasoned differential diagnosis in complex or uncertain cases.
**Diagnosis: Further Questions**	Asked a more thorough and targeted set of questions, covering lifestyle, medications, and a wider range of symptoms.	Asked specific, insightful questions related to patient history (e.g., symptoms at a young age) that suggested a different clinical focus.
**Diagnosis: Further Tests**	Recommended a more modern and comprehensive testing strategy, frequently including genetic testing, CPET/CPX, and advanced imaging.	Suggested essential, standard-of-care tests (e.g., CMR) that were sometimes missed by the other group.
**Management Plan**	Formulated more comprehensive and guideline-driven plans that included advanced therapies, ICD consideration, and family screening.	Demonstrated better clinical judgment in specific instances, such as correctly applying anticoagulation guidelines or avoiding premature medication recommendations.

SI.5.2 AI Analysis of subspecialists' individual evaluation comments

Gemini 2.5 Pro Analysis of Individual Subspecialist Evaluation Comments

Overall Summary of Preference. The analysis reveals distinct performance profiles for the two groups. The AI-assisted cardiologists produced more comprehensive responses, with significantly fewer omissions of key diagnostic tests and modern screening protocols (e.g., genetic testing). However, their responses were more prone to diagnostic overreach, excessive detail ("info dumps"), and occasional leaps in logic that were flagged by reviewers.
Conversely, the unassisted cardiologists were generally praised for clear, concise, and logical reasoning. Their weakness lay in significant omissions, particularly in missing modern standards of care like genetic/family screening and specific diagnostic workups. They were also flagged for more errors related to fundamental management decisions (medications, devices) and for suggesting unnecessary invasive procedures.
In essence, the AI-assisted group demonstrates breadth at the risk of focus, while the unassisted group shows focus at the risk of completeness.

Clinically Significant Errors. This dimension captures mistakes that could directly impact patient outcomes. The unassisted group had a higher volume of errors pointed out (22 vs. 15), with a notable concentration in missed diagnoses and incorrect management of medications and devices. Unassisted Trend: Errors were frequently related to overlooking potential diagnoses (especially genetic conditions) and fundamental management steps like GDMT, ICD consideration, and anticoagulation. AI-Assisted Trend: Errors were more often related to misinterpreting specific findings (e.g., suggesting Mavacamten without LVOTO), over-focusing on one diagnosis while missing another (e.g., amyloid vs. HCM), and missing key patient history details.

Extra Content (Unnecessary/Inappropriate). This dimension assesses suggestions for tests, diagnoses, or information that are not indicated, low-yield, or inappropriate. Both groups had an equal number of flags (17), but the nature of the extra content differed significantly. Unassisted Trend: Feedback focused on suggestions for unnecessary invasive procedures or high-cost tests (e.g., cath, EP study, endomyobiopsy). AI-Assisted Trend: Feedback centered on diagnostic overreach (speculating on rare conditions) and excessive verbosity ("info dump," "too many details"), suggesting the AI may be providing an exhaustive but clinically unfocused list of possibilities.

Missing Content (Omissions). This dimension highlights the most significant difference between the two groups. The unassisted group was flagged for omitting critical information far more frequently than the AI-assisted group (30 vs. 19 instances). Unassisted Trend: A major pattern of missing modern screening protocols, with 10 distinct mentions of missed genetic or family screening. Omissions were also common across diagnostic workups and therapeutic considerations. AI-Assisted Trend: While still having omissions, this group was more consistently comprehensive. The omissions were less concentrated in a single area, suggesting more sporadic oversights rather than a systemic gap in knowledge about modern screening.

Correct Reasoning Steps. This dimension evaluates the logical flow and clarity of the response. Both groups were overwhelmingly praised for their reasoning. Unassisted Trend: Commended for clear, logical, and direct reasoning. AI-Assisted Trend: Also praised for clarity, but received twice as many explicit criticisms for "leaps in logic" or "jumping to conclusions," which aligns with the findings in the "Extra Content" category.

Figure SI.7 | Gemini 2.5 Pro analysis of subspecialist individual evaluation comments.

Table SI.8 | Gemini 2.5 Pro's analysis of subspecialists' comments: Counts of different error types per evaluation dimension. Gemini 2.5 pro was asked to categorize comments within each of the four dimensions and compare the frequencies of errors for unassisted and AI-assisted cardiologists. Illustrative examples of the different error types are shown in Table SI.9

**Dimension**	**Unassisted Cardiologists**	**AI-Assisted Cardiologists**
Clinically Significant Errors	Total Comments: 22<br>• Missed/Incorrect Diagnosis or DDx: 7<br>• Management Errors (Meds/Device): 7<br>• Screening/Testing Errors: 5<br>• Flawed Diagnostic Process: 3	Total Comments: 15<br>• Missed/Incorrect Diagnosis or DDx: 5<br>• Flawed Diagnostic Process: 4<br>• Screening/Testing Errors: 4<br>• Management Errors (Meds/Device): 2
Extra Content	Total Comments: 17<br>• Inappropriate Meds/Management: 7<br>• Unnecessary Procedures/High-Cost Tests: 6<br>• Diagnostic Overreach/Speculation: 4	Total Comments: 17<br>• Diagnostic Overreach/Speculation: 7<br>• Excessive Detail / Irrelevant History: 5<br>• Unnecessary Procedures/High-Cost Tests: 3<br>• Inappropriate Meds/Management: 2
Missing Content	Total Comments: 30<br>• Missed Screening/Genetics: 10<br>• Missed Diagnostic Workup: 9<br>• Incomplete DDx/History: 6<br>• Missed Management/Therapeutics: 5	Total Comments: 19<br>• Missed Diagnostic Workup: 7<br>• Missed Screening/Genetics: 5<br>• Incomplete DDx/History: 4<br>• Missed Management/Therapeutics: 3
Correct Reasoning Steps	Total Comments: 32<br>• Praised for Clarity/Logic: 30<br>• Criticized for Leaps in Logic: 2	Total Comments: 32<br>• Praised for Clarity/Logic: 28<br>• Criticized for Leaps in Logic: 4

Table SI.9 | Illustrative examples across Gemini 2.5 Pro's taxonomy for subspecialists' comments.

Taxonomy Item	Unassisted Cardiologist Examples	AI-Assisted Cardiologist Examples
Category: Clinically Significant Errors		
Missed/Incorrect Diagnosis or DDx	"Jumps to conclusions quickly on arrhythmogenic cardiomyopathy."<br>"HCM not correct diagnosis. Didnt ask about social history for DCM."	"Narrowed in on amyloid and did not consider HCM..."<br>"Misses hypertrabeculation."
Management Errors (Meds/Device)	"Missing considering anticoagulation for Afib is an error."<br>"Didnt say ACM, also didnt start GDMT or consider ICD."	"Mentions LVOTO and mavacamten when there is not an LVOTO."
Screening/Testing Errors	"Forgot family screening."<br>"Not clearly offering genetic testing after diagnosing HCM."	"Lack of genetic testing."<br>"Forgetting family screening."
Flawed Diagnostic Process	"didnt ask about syncope."	"age is incorrect in their summary."<br>"Missing adriamycin history."
Category: Extra Content		
Unnecessary Procedures / High-Cost Tests	"EP study likely not necessary."<br>"FDG-PET not necessary."	"Not sure amyloid and sarcoid are necessary work ups."<br>"VO2 testing as noted... Also SPEP low utility."
Diagnostic Overreach / Speculation	"Dx of amyloid is a bit much at this stage."<br>"presumptive dx of long qt a stretch at this time."	"Jumping to LVNC is a bit much at this stage."<br>"Probably a stretch to say this patient has a genetic..."
Inappropriate Meds / Management	"Not sure that DOAC is indicated for 'any' arrhythmia..."	"No indication to start GDMT."
Excessive Detail / Irrelevant History	N/A	"Quite the info dump."<br>"Goes into too much detail imho."
Category: Missing Content		
Missed Screening / Genetics	"Should order genetic testing, not consider ordering."<br>"Missed genetic testing and zio."	"Genetic testing."<br>"Family screening."
Missed Diagnostic Workup	"coronary evaluation."<br>"didnt do PYP for amyloid."	"Should have ruled out CAD."<br>"Why does this patient have wmas? Should have a workup."
Incomplete DDx / History	"DDx is limited (jumps to catecholamine PMVT)."<br>"Social Hx. Mentioning DCM as most likely diagnosis."	"Could have offerred more DDx."<br>"It should have included HCM in the DDx."
Missed Management / Therapeutics	"Needed to mention mava."<br>"Could have considered GDMT for low LVEF."	"No medications offerred for HCM (e.g. beta-blockers)."<br>"could have mentioned ICD stratification."
Category: Correct Reasoning Steps		
Praised for Clarity / Logic	"clear logical flow and clarity."<br>"Correct diagnosis -> correct management."	"Normal tests -> focused on preventative care."<br>"Identifies aorta and appropriate follow up..."
Criticized for Leaps in Logic	"Jumps to conclusions quickly..."	"The ischemic cardiomyopathy comes out of nowhere."<br>"Quite the logical leap to go from RSR'..."
