# Supplementary material for: A large language model for complex cardiology care
Source: Nat Med. 2026 Feb 6;32(2):616–23. doi: 10.1038/s41591-025-04190-9 (PMC12920087; doi:10.1038/s41591-025-04190-9)
Supplement: Supplementary file 2 — Reporting Summary [file 41591_2025_4190_MOESM2_ESM.pdf]

nature portfolio

Corresponding author(s): Tao Tu, Euan Ashley

Last updated by author(s): 09/29/2025

Reporting Summary

Nature Portfolio wishes to improve the reproducibility of the work that we publish. This form provides structure for consistency and transparency in reporting. For further information on Nature Portfolio policies, see our Editorial Policies and the Editorial Policy Checklist.

Please do not complete any field with "not applicable" or n/a. Refer to the help text for what text to use if an item is not relevant to your study.
For final submission: please carefully check your responses for accuracy; you will not be able to make changes later.

Statistics

For all statistical analyses, confirm that the following items are present in the figure legend, table legend, main text, or Methods section.

n/a	Confirmed	
☐	☒	The exact sample size (n) for each experimental group/condition, given as a discrete number and unit of measurement
☐	☒	A statement on whether measurements were taken from distinct samples or whether the same sample was measured repeatedly
☐	☒	The statistical test(s) used AND whether they are one- or two-sided<br>*Only common tests should be described solely by name; describe more complex techniques in the Methods section.*
☒	☐	A description of all covariates tested
☒	☐	A description of any assumptions or corrections, such as tests of normality and adjustment for multiple comparisons
☐	☒	A full description of the statistical parameters including central tendency (e.g. means) or other basic estimates (e.g. regression coefficient) AND variation (e.g. standard deviation) or associated estimates of uncertainty (e.g. confidence intervals)
☐	☒	For null hypothesis testing, the test statistic (e.g. F, t, r) with confidence intervals, effect sizes, degrees of freedom and P value noted<br>*Give P values as exact values whenever suitable.*
☒	☐	For Bayesian analysis, information on the choice of priors and Markov chain Monte Carlo settings
☒	☐	For hierarchical and complex designs, identification of the appropriate level for tests and full reporting of outcomes
☒	☐	Estimates of effect sizes (e.g. Cohen's d, Pearson's r), indicating how they were calculated

Our web collection on statistics for biologists contains articles on many of the points above.

Software and code

Policy information about availability of computer code

Data collection	The algorithms and scripts were implemented using Python 2.7.18 for data collection.
Data analysis	The data analysis scripts were implemented in Python 2.7.18. We are unable to release code used for analysis but describe these methods fully.

For manuscripts utilizing custom algorithms or software that are central to the research but not yet described in published literature, software must be made available to editors and reviewers. We strongly encourage code deposition in a community repository (e.g. GitHub). See the Nature Portfolio guidelines for submitting code & software for further information.

Data

Policy information about availability of data

All manuscripts must include a data availability statement. This statement should provide the following information, where applicable:

- Accession codes, unique identifiers, or web links for publicly available datasets
- A description of any restrictions on data availability
- For clinical datasets or third party data, please ensure that the statement adheres to our policy

Data consists of clinical test text data (ECGs, CMRs, rest and stress TTEs, ambulatory holter monitors, cardiopulmonary stress tests). All data is available open-sourced, available at https://redivis.com/datasets/1z3x-2354972da?v=next. Data is licensed under open-source license CC 4.0.

Research involving human participants, their data, or biological material

Policy information about studies with human participants or human data. See also policy information about sex, gender (identity/presentation), and sexual orientation and race, ethnicity and racism.

Reporting on sex and gender	We do not provide subanalysis for sex or gender. We are evaluating respondents on a representative set of consecutive patients from a single center. Our study design was not optimized for more nuanced sub-analyses (low sample size).
Reporting on race, ethnicity, or other socially relevant groupings	We do not report on or use race, ethnicity, or other socially relevant groupings.
Population characteristics	Mean age: 59, range (18-96). We list further characteristics of the patient data in Table 1 including availability of each type of clinical text data and the distribution of their diagnoses.
Recruitment	Data from 107 consecutive patients at Stanford was used. This population may not be fully representative of those with potential genetic cadiovascular disease as it is a single center that specializes in inherited cardiovascular disease, however, we describe the observed distribution of patient diagnoses in Table 1 and describe limitations of single center in the discussion. .
Ethics oversight	The clinical subspecialist evaluator component of this research involved the participation of physicians. This study adhered to the principles outlined in the Declaration of Helsinki. Informed consent was obtained from each physician before their participation. This study used only retrospective, de-identified data and was deemed to fall outside the scope of institutional review board oversight.

Note that full information on the approval of the study protocol must also be provided in the manuscript.

Field-specific reporting

Please select the one below that is the best fit for your research. If you are not sure, read the appropriate sections before making your selection.

☒ Life sciences ☐ Behavioural & social sciences ☐ Ecological, evolutionary & environmental sciences

For a reference copy of the document with all sections, see nature.com/documents/nr-reporting-summary-flat.pdf

Life sciences study design

All studies must disclose on these points even when the disclosure is negative.

Sample size	Sample size (107) was primarily constrained by cost and availability of cardiologist and subspecialist assessments. The sample size sufficient for the planned analyses. A minimal additional set (9) was used for domain specialization of the model.
Data exclusions	No data was excluded.
Replication	We use a pool of multiple general cardiologists to assess cases, and a pool of multiple subspecialists to evaluate to combat the inherent variability in human ratings. We also open-source the dataset to encourage replication.
Randomization	During subspecialist evaluation, the order of the assessments they were provided to grade was randomized and blinded.<br>The pool of cardiologists were randomized to manage these cases either with or without assistance from AMIE.
Blinding	Subspecialists were blinded to the source of the assessments that they graded.

Reporting for specific materials, systems and methods

We require information from authors about some types of materials, experimental systems and methods used in many studies. Here, indicate whether each material, system or method listed is relevant to your study. If you are not sure if a list item applies to your research, read the appropriate section before selecting a response.

Materials & experimental systems

n/a	Involved in the study
☒	☐ Antibodies
☒	☐ Eukaryotic cell lines
☒	☐ Palaeontology and archaeology
☒	☐ Animals and other organisms
☐	☒ Clinical data
☒	☐ Dual use research of concern
☒	☐ Plants

Methods

n/a	Involved in the study
☒	☐ ChIP-seq
☒	☐ Flow cytometry
☒	☐ MRI-based neuroimaging

Clinical data

Policy information about clinical studies

All manuscripts should comply with the ICMJE guidelines for publication of clinical research and a completed CONSORT checklist must be included with all submissions.

Clinical trial registration	The full clinical trial protocol is available on clinicaltrials.gov (NCT06935253).
Study protocol	General cardiologists will be randomized to manage real patient cases from a cardiovascular genetic cardiomyopathy clinic, with or without AI assistance.
Data collection	The study used routinely collected, retrospective, de-identified health data from a large academic medical center in the United States. The clinical data spanned the period from January 2022 to December 2023. General cardiologists, who served as research participants, were recruited between January 2025 and March 2025.
Outcomes	The primary and secondary outcomes were predefined in line with prior work (Goh Nature Medicine volume 31, pages1233–1238 (2025)) and a priori expert consensus. Outcomes were measured blindly and automatically through subspeciality preference and general cardiologists.

Plants

Seed stocks	N/A
Novel plant genotypes	N/A
Authentication	N/A

This checklist template is licensed under a Creative Commons Attribution 4.0 International License, which permits use, sharing, adaptation, distribution and reproduction in any medium or format, as long as you give appropriate credit to the original author(s) and the source, provide a link to the Creative Commons license, and indicate if changes were made. The images or other third party material in this article are included in the article's Creative Commons license, unless indicated otherwise in a credit line to the material. If material is not included in the article's Creative Commons license and your intended use is not permitted by statutory regulation or exceeds the permitted use, you will need to obtain permission directly from the copyright holder. To view a copy of this license, visit http://creativecommons.org/licenses/by/4.0/
